# Dying tumor cell-derived exosomal miR-194-5p potentiates survival and repopulation of tumor repopulating cells upon radiotherapy in pancreatic cancer

**DOI:** 10.1186/s12943-020-01178-6

**Published:** 2020-03-30

**Authors:** Ming-jie Jiang, Yi-yun Chen, Juan-juan Dai, Dian-na Gu, Zhu Mei, Fu-rao Liu, Qian Huang, Ling Tian

**Affiliations:** 1 Institute of Translational Medicine, Shanghai General Hospital, Shanghai Jiao Tong University School of Medicine, Shanghai, 201620 China; 2 Shanghai Key Laboratory of Pancreatic Diseases, Shanghai General Hospital, Shanghai Jiao Tong University School of Medicine, Shanghai, 201620 China; 3 Department of Chemoradiotherapy, The First Affiliated Hospital of Wenzhou Medical University, Zhejiang, 325000 China; 4 Cancer Center, Shanghai General Hospital, Shanghai Jiao Tong University School of Medicine, Shanghai, 201620 China; 5 Department of Central Laboratory, Shanghai Chest Hospital, Shanghai Jiao Tong University School of Medicine, Shanghai, 200030 China

**Keywords:** Tumor repopulation, Tumor repopulating cell, Exosome, microRNA, DNA damage response, Aspirin, Radiotherapy, Pancreatic cancer

## Abstract

**Background:**

Tumor repopulation is a major cause of radiotherapy failure. Previous investigations highlighted that dying tumor cells played vital roles in tumor repopulation through promoting proliferation of the residual tumor repopulating cells (TRCs). However, TRCs also suffer DNA damage after radiotherapy, and might undergo mitotic catastrophe under the stimulation of proliferative factors released by dying cells. Hence, we intend to find out how these paradoxical biological processes coordinated to potentiate tumor repopulation after radiotherapy.

**Methods:**

Tumor repopulation models in vitro and in vivo were used for evaluating the therapy response and dissecting underlying mechanisms. RNA-seq was performed to find out the signaling changes and identify the significantly changed miRNAs. qPCR, western blot, IHC, FACS, colony formation assay, etc. were carried out to analyze the molecules and cells.

**Results:**

Exosomes derived from dying tumor cells induced G1/S arrest and promoted DNA damage response to potentiate survival of TRCs through delivering miR-194-5p, which further modulated E2F3 expression. Moreover, exosomal miR-194-5p alleviated the harmful effects of oncogenic HMGA2 under radiotherapy. After a latent time, dying tumor cells further released a large amount of PGE2 to boost proliferation of the recovered TRCs, and orchestrated the repopulation cascades. Of note, low-dose aspirin was found to suppress pancreatic cancer repopulation upon radiation via inhibiting secretion of exosomes and PGE2.

**Conclusion:**

Exosomal miR-194-5p enhanced DNA damage response in TRCs to potentiate tumor repopulation. Combined use of aspirin and radiotherapy might benefit pancreatic cancer patients.

## Introduction

Cancer radiotherapy is one of the main strategies of cancer treatment, and more than half of cancer patients receive radiotherapy to cure localized disease, palliate symptoms, or control disease in incurable cancers [1]. However, in a small set of cancers such as pancreatic cancer, the effects of radiotherapy remain unsatisfactory [2], which contributes to the fact that pancreatic cancer ranks one of the most malignant cancers with the lowest 5-year survival rate among all kinds of cancers [3]. As one of the “5Rs” of radiobiology, tumor repopulation is taken for a major cause of treatment failure [4]. Our previous works revealed that tumor repopulation significantly occurred in pancreatic cancer after radiation [5–7]. Nevertheless, its underlying mechanisms largely remain unclear.

Recent researches showed that chemoradiation-caused dying tumor cells promoted tumor repopulation [5, 8]. The irradiated dying tumor cells underwent significant intracellular signaling changes and secreted large amounts of molecules. It was reported that the lipid metabolite PGE2 secreted by dying tumor cells promoted tumor repopulation [8]. Moreover, dying tumor cells also secreted HMGB1 [9], VEGF [10], etc. to accelerate proliferation of the admixed surviving tumor cells, promote angiogenesis, and facilitate cancer cell repopulation and metastasis [5].

As known, the secretions of dying tumor cells are complex, and usually comprise cytoplasmic soluble factors, organelles and their contents, and extracellular vesicles (EVs) [9, 11, 12]. Notably, EVs, especially exosomes, are important mediators of cell-cell communications and play vital roles in promoting cancer progression [13]. Moreover, microRNAs (miRNAs), a type of small non-coding RNA, are vital contents of EVs. Loading of miRNAs into exosomes is not a random process, and the selective enrichment of miRNAs are tightly regulated such as by *KRAS* mutation [14]. Cancer cell-secreted exosomal miRNAs were found to involve in stroma cell reprogramming [15] and pre-metastatic niche formation [16]. And exosomal miRNAs from cancer stroma cells were reported to confer chemoresistance [17]. However, little is known about whether exosomes are involved in tumor repopulation.

Besides, tumor repopulating cells (TRCs) are supposed to be the major cells for tumor repopulation, and exert some cancer stem-like cell (CSC) properties [18, 19]. Yet, TRCs just represent a functional distinction. It is still unclear whether there’re any relative markers to define TRCs. Some markers, such as c-MET, CD44, CD133, LGR5, ALDH1, etc. are used to identify pancreatic CSCs [20]. Do these markers also apply to identifying TRCs in pancreatic cancer? Further, TRCs also suffer from radiation as dying cells do and sustain DNA damages in radiotherapy. Cell cycle progression with DNA damage could induce mitotic catastrophe, which is the main form of cell death induced by ionizing radiation (IR) [21]. Considering that large amounts of proliferation stimuli are released by irradiated dying tumor cells, it is important to figure out how TRCs survive and are stimulated to fast proliferation under IR-induced damages. This study aims to delineate how TRCs survive and repopulate after radiotherapy, and seeks appropriate agents to intervene pancreatic cancer repopulation.

Herein, we first reported that exosomal miR-194-5p derived from radiation-caused dying tumor cells potentiated tumor repopulation. We found that irradiated dying tumor cells released a large amount of exosomes in the early phase after radiation. These exosomes further enhanced DNA damage responses to promote survival of TRCs that were characterized as ALDH1^+^ cells. Next generation sequencing of exosomal miRNAs identified miR-194-5p as the significantly elevated miRNA. We further found that miR-194-5p could downregulate the transcription factor E2F3 to induce cell cycle arrest and contribute to repairing the damaged TRCs. Moreover, the transfer of miR-194-5p to TRCs might alleviate the harmful effects of HMGA2 under radiotherapy. Subsequently, dying tumor cells released PGE2 to accelerate proliferation of the repaired TRCs. More importantly, low-dose aspirin was found to suppress tumor repopulation via inhibiting the secretion of exosomes and PGE2. Our data provides new insights into tumor repopulation and suggests a simple and feasible intervention method for pancreatic cancer.

## Results

### Exosomes released from irradiated dying tumor cells dynamically potentiate tumor repopulation

We firstly examined the biological processes of tumor repopulation after radiotherapy. In tumor repopulation model in vitro, where irradiated dying tumor cells (feeder cells) were co-cultured with the admixed living tumor cells (reporter cells) as described before [8], we found that dying tumor cells accelerated proliferation of reporter cells after 3 days (Additional file 1: Fig. S1a). However, the proliferation of reporter cells was slightly inhibited when co-cultured for 1 day (Additional file 1: Fig. S1b). To further clarify the timepoint of cell cycle changes, we applied a transwell co-culture system, where the upper layer was plated with irradiated or unirradiated feeder cells and the lower layer was viable reporter cells. It was showed that irradiated dying tumor cells induced G1/S cell-cycle arrest in reporter cells 12 h after radiation, but the effect was reversed 36 h after radiation (Additional file 1: Fig. S1c). Further, when reporter cells were cultured with conditioned media (CM) from irradiated dying tumor cells, similar proliferation-inhibitory effects were shown (Additional file 1: Fig. S1d). These data indicate that secretions from irradiated dying tumor cells inhibit cell proliferation at the immediate early phase after radiation but promote proliferation in the later phase.

**Fig. 1:**
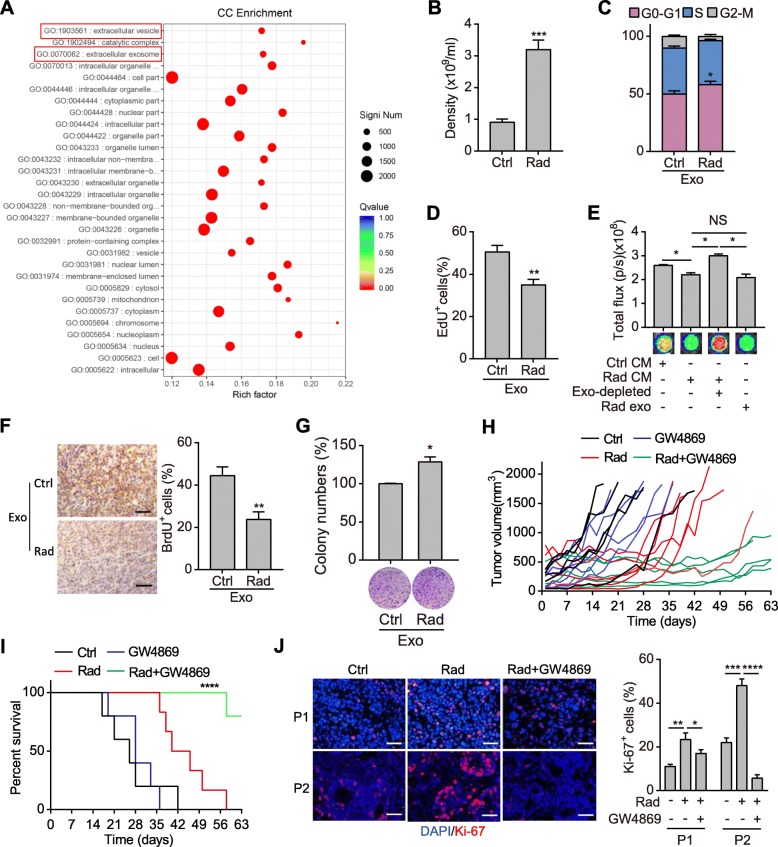
Exosomes released from irradiated dying tumor cells dynamically potentiate tumor repopulation. **a** Gene Ontology enrichment analysis of significantly changed mRNAs in SW1990 cells after radiation. **b** Density of exosomes isolated from the supernatant of unirradiated or 10Gy irradiated SW1990 cells. **c-d** Cell cycle assay (c) and EdU cell proliferation assay (d) of SW1990 cells treated with exosomes isolated from unirradiated or 10Gy irradiated SW1990 cells. **e** Cell viability analysis of SW1990 cells treated with CM of unirradiated or 10Gy irradiated SW1990 cells, or exosomes-depleted CM and the exosomes from irradiated cells, respectively. **f** Representative images (left) and quantifications (right) of IHC staining of BrdU in SW1990 subcutaneous tumors treated with exosomes isolated from unirradiated or 10Gy irradiated SW1990 cells. Scale bar: 50 μm. **g** Colony formation assay of SW1990 cells. Cells were treated with exosomes derived from unirradiated or 10Gy irradiated SW1990 cells, and further subjected to 10Gy irradiation. Representative images (down) and quantifications (top) were shown. **h-i** Tumor growth curve (h) and survival rate (i) of the PDX tumor-bearing mice. The mice were treated with 10Gy radiation, GW4869. *n* = 5~6 for each group. **j** Representative images (left) and quantifications (right) of immunofluorescence staining of Ki-67 in PDX tissues treated with 10Gy radiation, or radiation plus GW4869. Scale bar: 50 μm. Data are presented as mean with SD of at least three independent experiments; ^*^p < 0.05; ^**^p < 0.01; ^***^p < 0.001; ^****^p < 0.0001 from unpaired Student’s t test

To figure out why dying tumor cells inhibited proliferation of reporter cells transiently after radiation, transcriptome sequencing of irradiated dying tumor cells was performed. Gene Ontology analysis revealed that signaling concerning EVs and exosomes regulation were enriched in irradiated cells (Fig. 1a; Additional file 2: Fig. S2a). We further confirmed the increased release of exosomes in CM of irradiated pancreatic cancer cells compared with unirradiated ones (Fig. 1b). These exosomes were well characterized by reference methods (Additional file 2: Fig. S2b-d). Moreover, proteins involving in exosome processing were also upregulated in irradiated cells as well as in patient-derived xenograft (PDX) mouse model of pancreatic cancer (Additional file 2: Fig. S2d-g). Further, dying tumor cell-derived exosomes were used to treat reporter cells. Exosomes from irradiated dying tumor cells induced G1/S cell cycle arrest, inhibited EdU incorporation and proliferation of the treated cells (Fig. 1c-e; Additional file 2: Fig. S2h-i). Moreover, exosomes-depleted CM of irradiated cells lost the inhibitory effects (Fig. 1e). In vivo administration of irradiated dying tumor cells-derived exosomes also suppressed proliferation of the subcutaneous tumors as indicated by BrdU incorporation assay (Fig. 1f). These data indicate that the proliferation inhibitory effect is largely due to dying cell-derived exosomes.

To verify the direct role of exosomes in surviving tumor cells, we performed colony formation assay in vitro. Colony formation ability of cancer cells treated with dying tumor cell-derived exosomes was significantly enhanced after radiation (Fig. 1g). Moreover, GW4869, an SMase inhibitor that was widely used for reducing exosome secretion [22], dramatically inhibited colony formation of pancreatic cancer cells after irradiation (Additional file 2: Fig. S2j). Meanwhile, GW4869 showed no effects on colony formation of the unirradiated cells (Additional file 2: Fig. S2k). These results indicate that exosomes derived from dying tumor cells can promote cell survival after radiation.

We further explored the role of exosomes in tumor repopulation in PDX mouse models. Irradiated PDX tumors showed accelerated repopulation after a lag phase, even though a little variance existed among tumors derived from different patients (Fig. 1h; Additional file 2: Fig. S2l-m). Surprisingly, when PDX mice were simultaneously treated with GW4869, tumor repopulation was significantly slowed down (Fig. 1h; Additional file 2: Fig. S2l-m), and the survival time of tumor-bearing mice was prolonged (Fig. 1i). Moreover, much more Ki-67^+^ cells resided in irradiated tumors, which was significant reduced by simultaneous GW4869 treatment (Fig. 1j). These results indicate that dying tumor cells-derived exosomes promote tumor repopulation.

### Dying tumor cells-released exosomes potentiate survival of the ALDH1A1^+^ TRCs

**Fig. 2:**
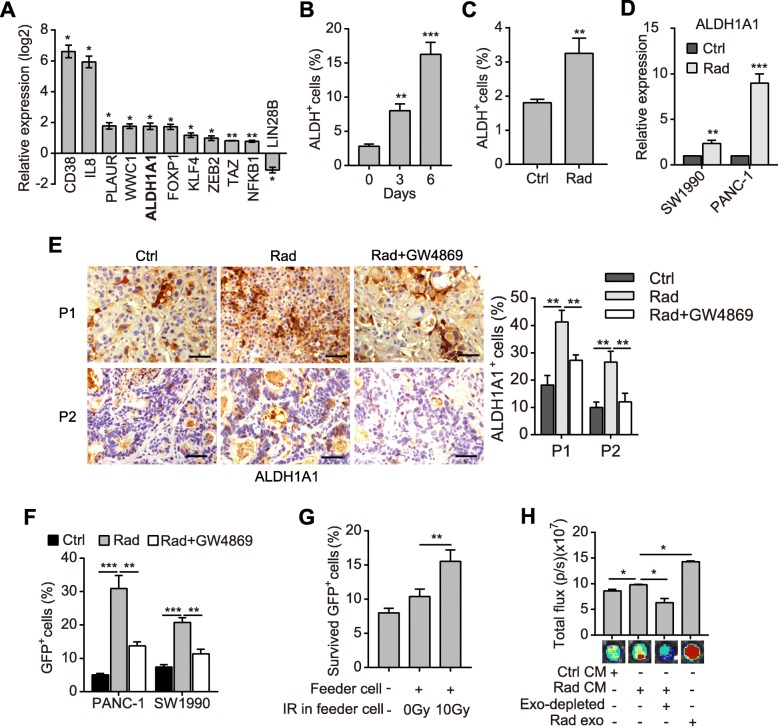
Dying tumor cell-released exosomes potentiate survival of the ALDH1A1^+^ TRCs. **a** Statistically significantly changed genes detected by qPCR array in 10Gy irradiated PANC-1 cells compared with unirradiated cells. **b** Percentage of ALDH^+^ cells in PANC-1 cells after 10Gy radiation. **c** Percentage of ALDH^+^ cells in unirradiated or 10Gy irradiated primary pancreatic cancer cells. **d** qPCR detection of ALDH1A1 in unirradiated or 10Gy irradiated SW1990 and PANC-1 cells. **e** Representative images (left) and quantifications (right) of IHC staining of ALDH1A1 in PDX tissues that were untreated, treated with radiation or radiation plus GW4869. Scale bar: 50 μm. **f** Percentage of GFP^+^ cells in SW1990 and PANC-1 tracing cells. Cells were induced with 4-OHT, 10Gy irradiated, and treated with or without GW4869. **g** Relative survival rate of the isolated GFP^+^ cells. GFP^+^ cells isolated from the tracing SW1990 cells were 10Gy irradiated and cultured alone, or co-cultured with irradiated or unirradiated pancreatic cancer cells. **h** Cell viability analysis of the isolated GFP^+^ cells. Irradiated GFP^+^ cells (as in Fig. 2g) were treated as in Fig. 1e. Data are presented as mean with SD of at least three independent experiments; ^*^p < 0.05; ^**^p < 0.01; ^***^p < 0.001 from unpaired Student’s t test

We supposed that TRCs might act as the primary recipients of exosomes. As currently no marker was applied to identify TRCs, we thus collected surviving pancreatic cancer cells after radiation and analyzed the expression profiles of cancer stem cell-related genes by qPCR array. It was identified that 62 of the 84 target genes were upregulated compared with the unirradiated controls (Additional file 3: Fig. S3a). Especially, 10 of the 11 statistically significantly changed genes were upregulated (Fig. 2a; Additional file 3: Fig. S3a). Moreover, the ratio of CD24^+^ cells and CD326 (ESA)^+^ cells were significantly upregulated in irradiated PANC-1 cells (Additional file 3: Fig. S3b). These data indicate that surviving cells exhibit higher CSC properties.

Remarkably, the expression of ALDH1A1 was significantly upregulated after radiation (Fig. 2a; Additional file 3: Fig. S3a). As known, ALDH1A1 was one of the most important markers of pancreatic cancer stem-like cells [23]. The ALDEFLOUR™ experiment revealed that the ratio of ALDH^+^ cells was significantly elevated in surviving cancer cells and primary pancreatic tumor cells after radiation (Fig. 2b-c; Additional file 3: Fig. S3c). qPCR assay and western blot demonstrated that the expression of ALDH1A1 was upregulated in irradiated pancreatic cancer cells and PDX tumor tissues (Fig. 2d; Additional file 3: Fig. S3d-e). Immunohistochemistry (IHC) staining further showed that ALDH1A1^+^ cells were highly enriched after irradiation in PDX tumor tissues (Fig. 2e). However, the accumulation of ALDH1A1^+^ cells was significantly inhibited by GW4869 (Fig. 2e), which indicated that exosomes might promote survival of the ALDH1A1^+^ cells.

To investigate how the ratio of ALDH1A1^+^ cells increased after irradiation, we constructed a lineage tracing system, in which GFP expression indicated ALDH1A1 expression after being induced by 4-hydroxytamoxifen (4-OHT) (Additional file 3: Fig. S3f). The ALDH1A1 expression was elevated in GFP^+^ cells (ALDH1A1^+^ cells) than in DsRed^+^ cells (ALDH1A1^−^ cells) (Additional file 3: Fig. S3g). The colony-forming capacity of GFP^+^ cells also improved in soft agar and in plate after irradiation compared with DsRed^+^ cells (Additional file 3: Fig. S3h-i). Notably, GFP^+^ cells were significantly enriched after irradiation, while additional treatment with GW4869 significantly suppressed the enrichment of GFP^+^ cells (Fig. 2f). Meanwhile, a higher proportion of GFP^+^ cells were also raised in subcutaneous tumors after radiation (Additional file 3: Fig. S3j).

Furthermore, we examined the direct protective role of dying tumor cells on TRCs. Irradiated GFP^+^ cells exhibited significantly higher survival rate when they were cocultured with irradiated pancreatic cancer cells or treated with CM thereof (Fig. 2g-h). As shown, exosome-depleted CM of irradiated cells lost the protective effect, while the isolated exosomes significantly promoted survival of irradiated GFP^+^ cells (Fig. 2h). These results indicate that the ALDH1A1^+^ cells represent a subpopulation of TRCs, whose survival is enhanced by dying tumor cell-derived exosomes under radiotherapy.

### Exosomes potentiate survival of TRCs via promoting DNA damage response

**Fig. 3:**
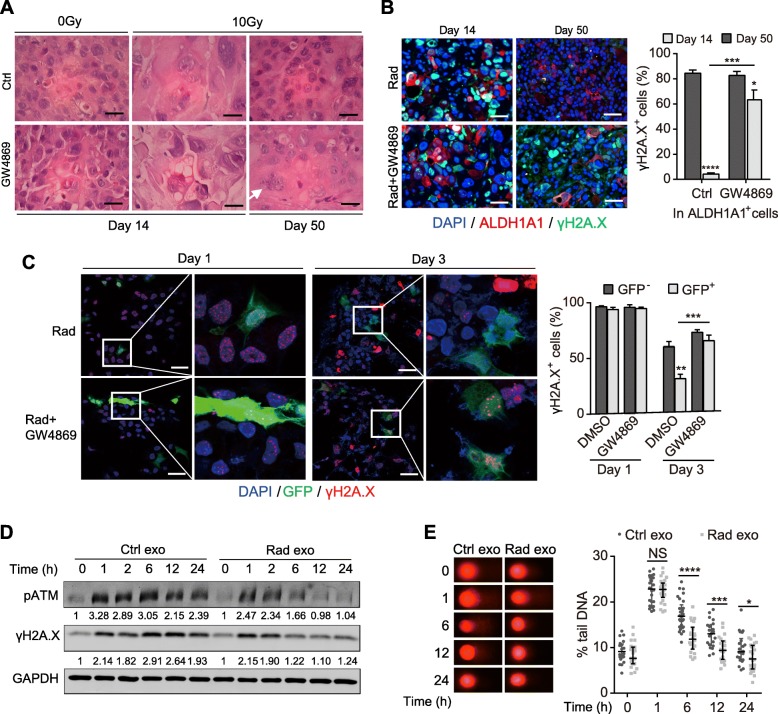
Exosomes potentiate survival of TRCs via promoting DDR. **a** Representative images of H&E staining of the PDX tissues that were untreated, or treated with GW4968, radiation or radiation plus GW4869. Arrow indicates the multinuclear giant cell. Scale bar: 20 μm. **b** Representative images (left) and quantifications (right) of immunofluorescent staining of ALDH1A1 (red) and γH2A.X (green) in PDX tumor tissues. Tumors were treated with radiation or radiation plus GW4869. Scale bar: 50 μm. **c** Representative images (left) and quantifications (right) of immunofluorescent staining of GFP (green) and γH2A.X (red) in the tracing PANC-1 cells. Cells were treated with radiation or radiation plus GW4869. Scale bar: 50 μm. **d** Western blot detection of pATM and γH2A.X in PANC-1 cells. Cells were treated with exosomes from irradiated or unirradiated cells, and subjected to 2Gy radiation. Numbers at the bottom of corresponding bands show a ratio of γH2A.X or pATM levels relative to time 0 after normalization to GAPDH. **e** Representative images (left) and quantifications (right) of comet assay in PANC-1 cells. Cells were treated as in Fig. 3d. Data are presented as mean with SD of at least three independent experiments; ^*^p < 0.05; ^**^p < 0.01; ^***^p < 0.001; ^****^p < 0.0001; NS, not significant from unpaired Student’s t test

We then explored how dying tumor cell-derived exosomes promoted survival of the ALDH1A1^+^ cells. H&E staining showed that cells in the PDX tumors presented enlarged nuclei with significant heteromorphosis 14 days after radiation (Fig. 3a), which indicated the accumulation of radiation damage. 50 days after radiation, cells recovered, and “normal” cell nuclei morphology was returned. However, the recovery of tumor cells was hindered by GW4869 (Fig. 3a). Notably, ALDH1A1^+^ cells also suffered from DNA damage after radiation as indicated by the double staining of ALDH1A1 and γH2A.X (Fig. 3b). And in the GW4869 treatment group, majority of ALDH1A1^+^ cells still manifested significant γH2A.X positive foci 50 days after radiation (Fig. 3b). These data indicate that the survival of TRCs after radiation involves DNA damage response (DDR), which is modulated by exosomes from irradiated dying tumor cells.

Further, we testified whether exosomes directly promote DDR for survival of irradiated TRCs in vitro. As showed in the tracing cells, irradiation caused DNA damage in both GFP^+^ and GFP^−^ cells (Fig. 3c). The DNA damages in GFP^+^ cells were rapidly repaired after radiation as evidenced by the faster elimination of γH2A.X positive foci. GW4869 significantly impaired the repair of GFP^+^ cells, for most of them still carried γH2A.X positive foci 3 days after radiation (Fig. 3c). Furthermore, exosomes derived from irradiated dying tumor cells significantly accelerated attenuation of γ-H2A.X and pATM (Fig. 3d) and reduction of DNA damage (Fig. 3e) in irradiated cells. Collectively, these results demonstrate that irradiated dying tumor cells-derived exosomes potentiate the survival of TRCs via promoting DDR.

### Exosomal miR-194-5p promotes repair of the damaged TRCs

**Fig. 4:**
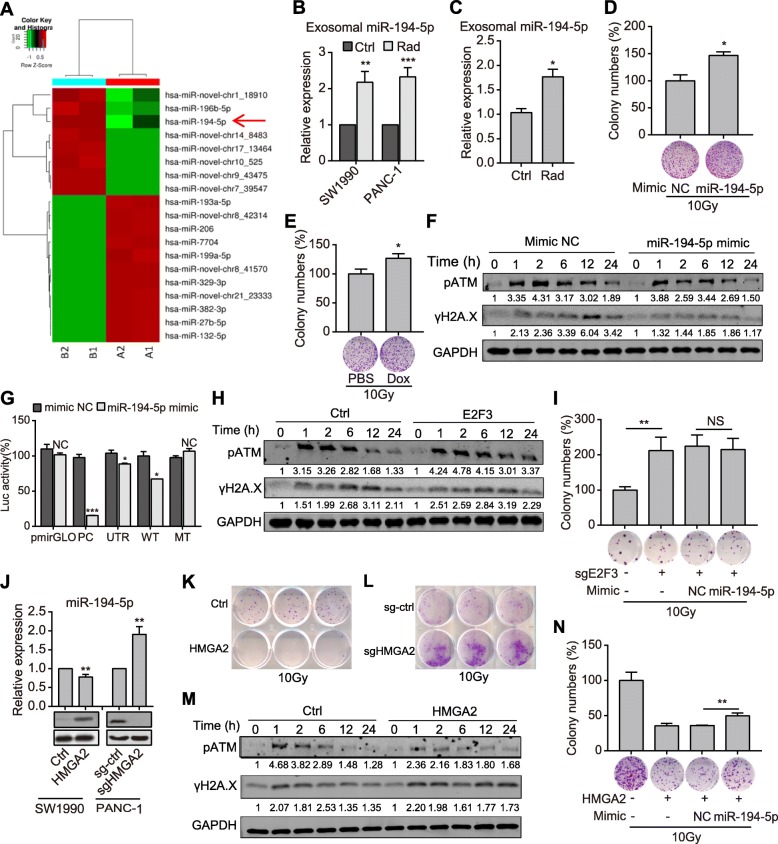
Exosomal miR-194-5p promotes repair of the damaged TRCs. **a** Heatmap of the significantly changed miRNAs in exosomes isolated from irradiated (group B) or unirradiated pancreatic cancer cells (group A). **b-c** qPCR detection of exosomal miR-194-5p. Exosomes were isolated from either irradiated/unirradiated cells (b) or plasma of PDX tumor-bearing mice (c). **d-e** Relative colony numbers (top) and representative images (down) of SW1990 cells (d) or SW1990 tet-on cells (e) that were treated as indicated. **f** Expression of pATM and γH2A.X in PANC-1 cells transfected with miR-194 mimics or mimic NC and subjected to 2Gy radiation. Numbers at the bottom of corresponding bands show a ratio of γH2A.X or pATM levels relative to time 0 after normalization to GAPDH. **g** Dual luciferase assay to identify E2F3 as the direct target of miR-194-5p in PANC-1 cells. The construction of reporter plasmids was shown in Fig. S9. **h** Expression of pATM and γH2A.X expression in the indicated PANC-1 cells treated with 2Gy radiation. Numbers are as described in Fig. 4f. **i** Colony formation assay of SW1990 cells that were treated as indicated. **j** qPCR analysis of the expression of miR-194-5p (top) and western blot analysis of the expression of HMGA2 (down) in pancreatic cancer cells. **k-l** Colony formation assay of the indicated SW1990 (k) and PANC-1(l) cells after 10Gy radiation. **m** Expression of pATM and γH2A.X in the indicated SW1990 cells treated with 2Gy irradiation. Numbers are as described in Fig. 4f. **n** Colony formation assay of SW1990 cells treated as indicated. Data are presented as mean with SD of at least three independent experiments; ^*^p < 0.05; ^**^p < 0.01; ^***^p < 0.001; NS, not significant from unpaired Student’s t test

As important cargos in exosomes, we inferred that miRNAs might play important roles in survival of the TRCs. So, we performed miRNA-seq and found that two validated miRNAs, miR-196b-5p and miR-194-5p, were significantly upregulated in dying tumor cells-derived exosomes (Fig. 4a). Interestingly, as a P53-induced miRNA, miR-194 might involve in genome stability [24]. miR-194-5p upregulation in exosomes derived from irradiated dying tumor cells was validated by qPCR assay (Fig. 4b). miR-194-5p expression was also upregulated in exosomes derived from the plasma of irradiated PDX mice (Fig. 4c), as well as in several pancreatic cancer cell lines after irradiation (Additional file 4: Fig. S4a).

Transfection of miR-194-5p mimics induced G1/S cell cycle arrest and suppressed EdU incorporation (Additional file 4: Fig. S4b-c), similar to the effect of exosomes from dying tumor cells. Moreover, stable overexpression of miR-194-5p significantly suppressed colony formation ability (Additional file 4: Fig. S4d). These results indicate that miR-194-5p suppresses pancreatic cancer cell proliferation. In addition, miR-194-5p overexpression also inhibited pancreatic cancer cell migration and invasion (Additional file 4: Fig. S4e-f).

However, miR-194-5p mimics-transfected cells exerted more powerful capabilities of colony formation after radiation (Fig. 4d). Further, a tet-on system was constructed to overexpress miR-194-5p under the control of doxycycline (Additional file 4: Fig. S4g). The colony formation of pancreatic cancer cells was dramatically inhibited when doxycycline was used (Additional file 4: Fig. S4h). However, if pancreatic cancer cells were exposed to radiation after doxycycline induction, colony formation of these cells would be significantly enhanced (Fig. 4e). Besides, miR-194-5p mimics significantly promoted attenuation of γH2A.X and pATM (Fig. 4f) and reduction of DNA damage (Additional file 4: Fig. S4i) in irradiated pancreatic cancer cells. These results indicate that exosomal miR-194-5p facilitates the survival of TRCs via promoting DDR.

We further identified the main effective targets of miR-194-5p. With the miRNA target prediction algorithms, 474 potential targets of miR-194-5p were predicted by Targetscan, 289 targets by Pictar, and amongst 87 targets were identical (Additional file 5: Fig. S5a). KEGG annotation revealed that only the transcription factor E2F3 in the predicted targets was related to cell cycle regulation (Additional file 5: Fig. S5a). E2F3, a member of E2F family, was widely revealed to promote cancer initiation and progression [25, 26]. The dual-luciferase reporter assay proved E2F3 as the direct target of miR-194-5p (Fig. 4g). E2F3 expression was inhibited by miR-194-5p mimics and miR-194-5p stably overexpression in pancreatic cancer cells, while slightly elevated by miR-194-5p inhibitor (Additional file 5: Fig. S5b-c). Contrary to miR-194-5p, E2F3 overexpression promoted cell cycle progression and EdU incorporation (Additional file 5: Fig. S5d-e), and hindered attenuation of pATM and γH2A.X (Fig. 4h) and reduction of DNA damage (Additional file 5: Fig. S5f) in irradiated pancreatic cancer cells. E2F3 knockout elevated the colony formation ability of pancreatic cancer cells after radiation, and transfection of miR-194-5p mimics didn’t further enhance the ability (Fig. 4i). These data indicate that exosomal miR-194-5p inhibits E2F3 to induce G1/S arrest and promote DNA damage repair.

Previous investigations identified HMGA2 and E2F3 as two of the eight key regulator hubs of pancreatic cancer [27]. The expression of HMGA2 was positively correlated with E2F3 in pancreatic cancer (Additional file 6: Fig. S6a). Unexpectedly, it was reported that HMGA2, an oncogene with pro-cancer stemness ability, interacted with miR-194 [28]. We found that HMGA2 negatively regulated miR-194-5p expression (Fig. 4j). HMGA2 and miR-194-5p were also revealed to be reversely correlated in pancreatic cancers by TCGA database analysis (Additional file 6: Fig. S6b).

HMGA2 expression was significantly upregulated in pancreatic cancer tissues compared with the normal pancreas tissues (Additional file 6: Fig. S6c). ALDH1A1^+^ pancreatic cancer cells also showed enhanced HMGA2 expression (Additional file 6: Fig. S6d). Meanwhile, HMGA2-overexpressed cells exhibited more stem cell-like features as shown by the higher proportion of ALDH^+^ cells (Additional file 6: Fig. S6e) and the greater ability of soft-agar colony formation (Additional file 6: Fig. S6f). HMGA2 overexpression also promoted proliferation of pancreatic cancer cells (Additional file 6: Fig. S6g), while HMGA2 knockout significantly inhibited their colony formation ability (Additional file 6: Fig. S6h). Besides, HMGA2 knockout significantly inhibited the migration and invasion of pancreatic cancer cells (Additional file 6: Fig. S6i-j). These data demonstrate that HMGA2 is enriched in the stem-like ALDH1A1^+^ cells and contributes to pancreatic cancer progression.

However, when pancreatic cancer cells were exposed to 10Gy radiation, overexpression of HMGA2 dramatically inhibited the colony formation ability of these cells (Fig. 4k), while knock-out of HMGA2 enhanced cell survival and colony formation (Fig. 4l). HMGA2 also hindered attenuation of pATM and γH2A.X (Fig. 4m) and reduction of DNA damage (Additional file 6: Fig. S6k) in irradiated pancreatic cancer cells. Importantly, the elevated HMGA2 expression in GFP^+^ cells was not significantly reduced after radiation (Additional file 6: Fig. S6l). Coincidentally, transfection of miR-194-5p in the HMGA2-overexpressed cancer cells partially enhanced the colony formation ability, and colonies of the miR-194-5p transfected group were significantly larger than that of the control (Fig. 4n). Together, these data indicate that the enriched HMGA2 in TRCs promotes their stemness and cancer progression but is potentially detrimental to the survival and recovery of irradiated TRCs, while exosomal miR-194-5p from dying tumor cells transiently reverses the effects of HMGA2 under radiotherapy to preserve the TRCs.

### Dying tumor cell-released PGE2 promotes proliferation of the recovered TRCs

**Fig. 5:**
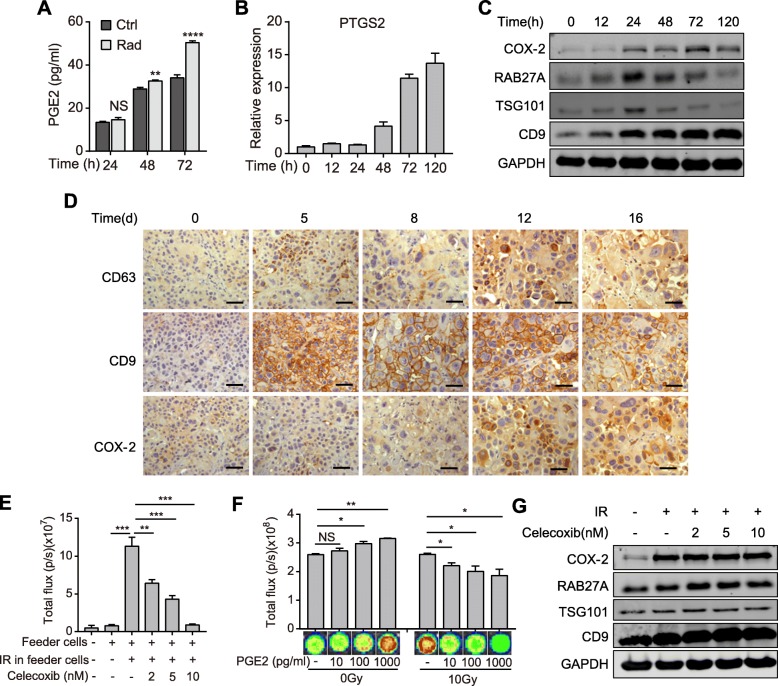
Dying tumor cell-released PGE2 promotes proliferation of the recovered TRCs. **a** ELISA detection of PGE2 contents in the supernatant of 10Gy irradiated or unirradiated SW1990 cells. **b** qPCR quantification of PTGS2 expression in SW1990 cells before and after 10Gy radiation. **c** Protein level of COX-2, RAB27A, TSG101 and CD9 in SW1990 cells before and after 10Gy radiation. **d** IHC staining of CD63, CD9 and COX-2 in PDX tumor tissue before and after 10Gy radiation. Scale bar: 50 μm. **e** Bioluminescence intensity of reporter cells that were cultured alone or co-cultured with feeder cells and treated with or without celecoxib as indicated. **f** Cell viability assay of PANC-1 cells treated with different concentration of PGE2 immediate after 10Gy/0Gy radiation. **g** Western blot detecting the expression of COX-2, RAB27A, TSG101 and CD9 in SW1990 cells treated with 10Gy radiation and different concentration of celecoxib. Data are presented as mean with SD of at least three independent experiments; ^*^p < 0.05; ^**^p < 0.01; ^***^p < 0.001; ^****^p < 0.0001; NS, not significant from unpaired Student’s t test

As described earlier, next to the proliferation inhibitory effects at the immediate early phase after radiation, dying tumor cells accelerated the proliferation of reporter cells (Additional file 1: Fig. S1), which implied that the recovered TRCs were driven to accelerate proliferation by some secretions other than aforementioned exosomal miR-194. As known, irradiated dying tumor cells also released PGE2, a lipid metabolite that was deemed to be a vital mediator of tumor repopulation after chemoradiation [8, 29]. Thus, we quantified PGE2 in the CM of irradiated dying pancreatic cancer cells by ELISA, and found that the contents of PGE2 remained unchanged in the CM at 24 h after radiation, yet slightly elevated at 48 h and significantly increased at 72 h (Fig. 5a). The change of PGE2 contents were in line with the proliferation dynamics of reporter cells cocultured with irradiated cells (Additional file 1: Fig. S1).

Corresponding to PGE2, the expression of PTGS2 (COX-2), a key enzyme for PGE2 biogenesis, also underwent a short lag phase before rapidly increased after radiation (Fig. 5b-c; Additional file 7: Fig. S7). In contrast, the expression of exosome-related proteins, such as RAB27A, TSG101 and CD9, increased immediately after radiation in pancreatic cancer cells (Fig. 5c; Additional file 7: Fig. S7b). Of note, when the expression of COX-2 gradually increased, the expression of exosome-related proteins decreased (Fig. 5c; Additional file 7: Fig. S7b). In the PDX mouse model, IHC staining also showed that the expression of exosome-related proteins was highly elevated soon after radiation till declined 12 days later, when the expression of COX-2 started to upregulate significantly (Fig. 5d).

Meanwhile, it was found that tumor cell repopulation was significantly restrained by celecoxib, a COX-2 inhibitor that inhibited PGE2 synthesis, in a dose-dependent manner (Fig. 5e). Although PGE2 was found to accelerate the proliferation of tumor cells, addition of PGE2 to tumor cells immediately after radiation reversely inhibited cell viability (Fig. 5f). Of note, celecoxib treatment didn’t influence the expression of proteins concerning exosomes processing (Fig. 5g). These results demonstrate that the secretions from irradiated dying tumor cells are released in a time-course manner, which are vital for tumor repopulation.

Together, these results indicate that dying tumor cell-released exosomes and PGE2 orchestrate tumor repopulation cascades, of which exosomal miR-194-5p firstly inhibits cell proliferation to promote recovery of TRCs, and then PGE2 boosts accelerated proliferation of the recovered TRCs.

### Aspirin suppresses tumor repopulation after radiotherapy via impairing the secretions from dying tumor cells

**Fig. 6:**
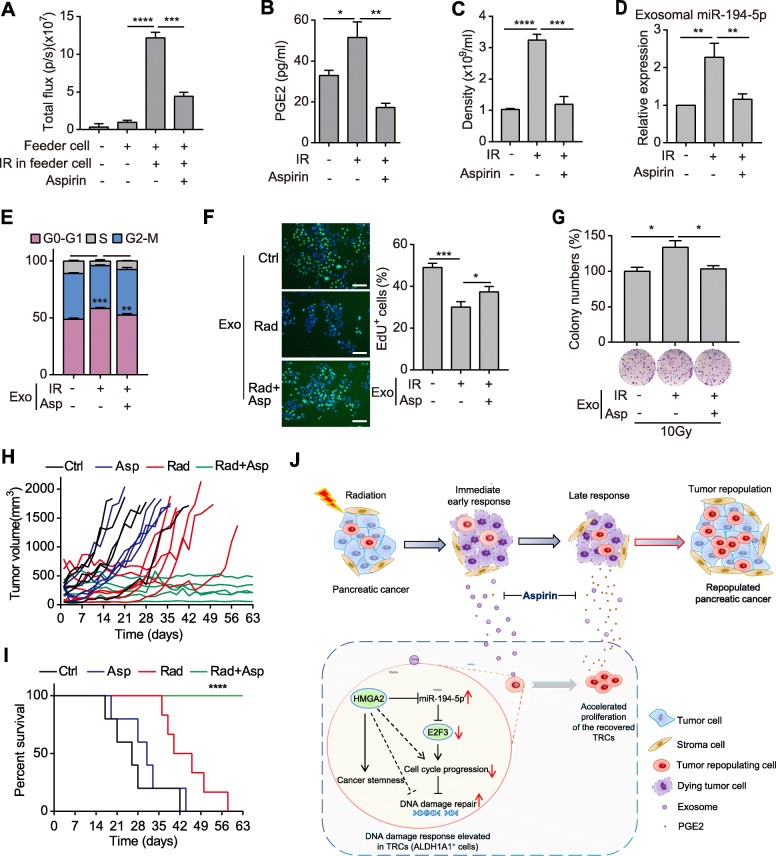
Aspirin suppresses tumor repopulation after radiotherapy via impairing the secretions from dying tumor cells. **a** Bioluminescence intensity of reporter cells that were cultured alone or co-cultured with feeder cells and treated with or without aspirin as indicated. **b** ELISA detecting the concentration of PGE2 in the supernatant of SW1990 cells that were treated with 10Gy irradiation or irradiation plus aspirin. **c** Density of exosomes released from SW1990 cells that were treated as in Fig. 6b. **d** qPCR detection of miR-194-5p in exosomes isolated from SW1990 cells treated as in Fig. 6b. **e-f** Cell cycle assay (e), representative images (left) and quantifications (right) of EdU incorporation (f) in SW1990 cells treated with exosomes from the indicated cells. Scale bar: 100 μm. **g** Relative colony numbers (top) and representative images (down) of SW1990 cells treated with exosomes from the indicated cells and subjected to 10Gy radiation. **h-i** Tumor growth curve (h) and survival rate (i) of PDX tumor-bearing mice that were untreated, or treated with aspirin, 10Gy radiation or radiation plus aspirin. *n* = 5~6 for each group. **j** Schematic diagram showing that aspirin disrupts dying tumor cell-orchestrated tumor repopulation cascades. Data are presented as mean with SD of at least three independent experiments; ^*^p < 0.05; ^**^p < 0.01; ^***^p < 0.001; ^****^p < 0.0001 from unpaired Student’s t test, expect in Fig. 6i, which was calculated by Log-rank (Mantel-Cox) test

Our previous discussion prompted that aspirin might have the potential to suppress tumor repopulation in pancreatic cancer [30]. And aspirin was also found to influence exosomes and miRNAs [31]. So, we firstly tested the effect of aspirin treatment in tumor cell repopulation model in vitro. Aspirin dramatically restrained the accelerated proliferation of reporter cells cocultured with irradiated dying tumor cells (Fig. 6a), while had no effect on the proliferation of reporter cells when they were cultured alone (Additional file 8: Fig. S8a). Aspirin reduced PGE2 contents in the supernatant of irradiated pancreatic cancer cells (Fig. 6b), and decreased the quantity of exosomes released from these cells (Fig. 6c). Besides, miR-194-5p was downregulated in exosomes derived from aspirin-treated irradiated cells (Fig. 6d). Moreover, compared with exosomes derived from cells treated with radiation alone, exosomes derived from the aspirin-treated irradiated cells lost the ability to induce G1/S arrest and reduce EdU incorporation (Fig. 6e-f). Accordingly, exosomes derived from the aspirin-treated irradiated cells couldn’t promote colony formation of pancreatic cancer cells after radiation (Fig. 6g).

We then tested the effects of aspirin treatment in PDX mouse models. Aspirin monotherapy had no therapeutic effects on PDX tumors, while radiotherapy alone showed marginally beneficial effects (Fig. 6h-i). However, combination of aspirin treatment and radiotherapy dramatically suppressed tumor repopulation and prolonged the survival time of tumor-bearing mice (Fig. 6h-i). Moreover, aspirin impaired the recovery of tumor tissues after radiotherapy (Additional file 8: Fig. S8b-c), which was similar to the effects of GW4869. Further, aspirin also suppressed the enrichment of ALDH1A1^+^ TRCs and reduced the proportion of Ki-67^+^ cells in tumor tissues after radiotherapy (Additional file 8: Fig. S8d-e). These data indicate that aspirin suppresses tumor repopulation after radiotherapy in pancreatic cancer.

## Discussions

Tumor repopulation is one of the “5Rs” of radiobiology and the main cause of treatment failure [4]. We herein propose that radiation-induced dying tumor cells orchestrate tumor repopulation. The irradiated dying cells firstly secret exosomes to induce cell cycle arrest and promote DNA damage repair to potentiate the survival of ALDH1A1^+^ TRCs, and then release large amounts of PGE2 to boost proliferation of the recovered TRCs. Low-dose aspirin could restrain secretion of exosomes and PGE2 from dying tumor cells, and suppress tumor repopulation after radiation in pancreatic cancer (Fig. 6j).

TRCs has been suggested to be the main cells responsible for tumor repopulation [18, 19]. However, TRCs also suffer from radiation in radiotherapy. As known, the main mechanism of radiotherapy is to induce DNA damage and cause cell death. The induction of DNA damage is accompanied by DDRs characterized by cell-cycle arrest and DNA damage repair. Moreover, the signaling of DDR such as P53 are mutated with high frequency in cancers, which has been exploited to enhance the efficacy of cytotoxic therapy or develop synthetic lethality strategies such as PARP inhibitors. Thus, to survive under radiotherapy, TRCs have to develop some additional mechanisms to compensate the impaired DDRs, such as getting help from dying cells.

Actually, it is a common phenomenon that dying cells promote survival of the tissue stem cells in different organisms. In Arabidopsis, chilling stress-induced programmed cell death in columella stem cell daughters protected stem cells through maintaining auxin maximum [32]. In Drosophila, irradiated dying cells were found to protect stem cells from death and keep them for regeneration, which was called the “Mahakali effect” [33]. In human, therapy-sensitive cells could promote survival and proliferation of the residue surviving tumor cells in targeted therapy [34]. We here have revealed that the “dying-for-surviving” phenomenon also exists in radiotherapy.

In addition to radiotherapy, other cytotoxic therapies such as chemotherapy or photodynamic therapy also enhanced the secretion of EVs from tumor cells [35]. EVs are also found to promote survival of stem cells by other studies. EV-packaged Wnt ligands derived from macrophage could rescue intestinal stem cells and enhance their survival after radiation [36]. Mesenchymal stromal cell-derived EVs also rescued radiation-damaged murine marrow hematopoietic cells via transferring miRNAs [37]. We here report that exosomes from irradiated dying tumor cells transfer miR-194-5p to the residual TRCs and facilitate their recovery. Nevertheless, it remains unclear why the secretion of exosomes elevates in irradiated dying tumor cells. Further investigations are needed.

Particularly, we here report that miR-194-5p is the mediator to enhance DDR and TRC survival in pancreatic cancer. Previous investigations also demonstrated that irradiated tumor cell-derived exosomes promoted radioresistance of the recipient cells via transferring miR-208a or miR-1246, which were found to promote cell proliferation in lung cancer [38, 39]. As far as we known, we are the first one to report that an exosome-transmitted proliferation inhibitory miRNA promotes radioresistance. As a P53-responsive miRNA, miR-194 was revealed to induce cell cycle arrest [24, 40]. CRISPR-Cas9 library-based screening revealed that miR-194-5p was a pro-fitness miRNA in Hela cells [41]. These data are in line with our findings. However, sustained cell cycle arrest may cause tumor suppression. Indeed, higher expression of miR-194-5p was associated with better prognosis in pancreatic cancer [42]. We also found that sustained overexpression of miR-194-5p inhibited cell proliferation, migration, invasion and colony formation. However, miR-194 was also reported to contribute to tumor growth and progression in pancreatic cancer [43]. Thus, the function of miR-194-5p might be highly context-dependent. Notably, exosomal miR-194-5p might performed like a “firefighter” in a way under radiotherapy, which was transferred from dying tumor cells to rescue TRCs in a short time. Once DNA damage was repaired, exosomal miR-194-5p was withdrawn, leaving no time for miR-194-5p to suppress the proliferation of TRCs. Accordingly, the lipid metabolite PGE2 were released from dying tumor cells, and took over to boost repopulation of the repaired TRCs. Moreover, exosomal miR-194 was also revealed to activate PMVECs and promote angiogenesis [44], which could potentiate tumor repopulation. Additionally, it was also found that HMGA2 attenuated DNA damage repair [45, 46], and E2F3 mediated DNA damage-induced apoptosis [47]. Our findings coincide with these data that both genes promote cancer progression but inhibit DNA damage repair.

Finally, low-dose aspirin was found to suppress tumor repopulation in pancreatic cancer after radiotherapy via inhibiting secretion of exosomes and PGE2 from dying tumor cells. These findings indicate that aspirin might have a good application prospect in radiotherapy. As an agent that has pleiotropic effects [30], aspirin might have some other mechanisms to repress tumor repopulation. Thus, more investigations should be carried out in the future to identify the specific approach of aspirin in repressing tumor repopulation. It was found that aspirin could reverse tumor-promoting inflammation and contribute to evoking tumor-inhibitory immunity [48], which indicated that aspirin might help to arouse the potential of immunogenic cell death, which could be induced by radiotherapy but often hindered [49, 50]. As a safe and widely available prescribed medicine, it is convenient to test the efficacy of aspirin in the clinic under appropriately designed clinical study.

## Conclusion

In summary, we found that dying tumor cells potentiated survival of the TRCs and promoted their repopulation in pancreatic cancer via sequentially secreting exosomes, which delivered miR-194-5p, and PGE2. Our data implied that combination treatment of radiotherapy and aspirin might greatly benefit pancreatic cancer patients.

## Supplementary information

**Additional file 1:Figure S1.** Irradiated dying tumor cells dynamically regulate proliferation of reporter cells.

**Additional file 2:Figure S2.** Increased secretion of exosomes from dying tumor cells inhibit cell proliferation but potentiate tumor repopulation.

**Additional file 3:Figure S3.** ALDH1A1^+^ cells are a subpopulation of TRCs with cancer stem cell-like properties.

**Additional file 4:Figure S4.** miR-194-5p suppresses pancreatic cancer cell proliferation, migration and invasion, but promotes DNA damage repair.

**Additional file 5:Figure S5.** E2F3 is a target of miR-194 and promotes pancreatic cell proliferation, inhibits DNA damage repair.

**Additional file 6:Figure S6.** HMGA2 promotes pancreatic cancer stemness and progression, but inhibits DNA damage repair.

**Additional file 7:Figure S7.** Expression of PTGS2 and exosome-related proteins in PANC-1 cells after radiation.

**Additional file 8:Fig. S8.** Effects and mechanisms of aspirin in suppressing pancreatic cancer repopulation.

**Additional file 9:Fig. S9.** Schematic diagram of the plasmid constructs.

**Additional file 10.** Supplementary materials and methods.

**Additional file 11:Table S1.** Oligos and primers used in this study.

## Data Availability

All data generated or analyzed during this study are included in this published article and its supplementary information files. The datasets generated during the current study are available in the GEO repository (GSE138983 and GSE138984).

## Abbreviations

4-OHT: 4-hydroxytamoxifen
CM: Conditioned media
CSC: Cancer stem-like cell
DDR: DNA damage response
EV: Extracellular vesicle
IHC: Immunohistochemistry
IR: Ionizing radiation
miRNA: microRNA
PDX: Patient-derived xenograft
TRC: Tumor repopulating cell
