# Supplementary material for: Dying tumor cell-derived exosomal miR-194-5p potentiates survival and repopulation of tumor repopulating cells upon radiotherapy in pancreatic cancer
Source: Mol Cancer. 2020 Mar 30;19:68. doi: 10.1186/s12943-020-01178-6 (PMC7104536; doi:10.1186/s12943-020-01178-6)
Supplement: Supplementary file 1 — Additional file 1:Figure S1. Irradiated dying tumor cells dynamically regulate proliferation of reporter cells. [file 12943_2020_1178_MOESM1_ESM.pdf]

Supplementary Figure S1

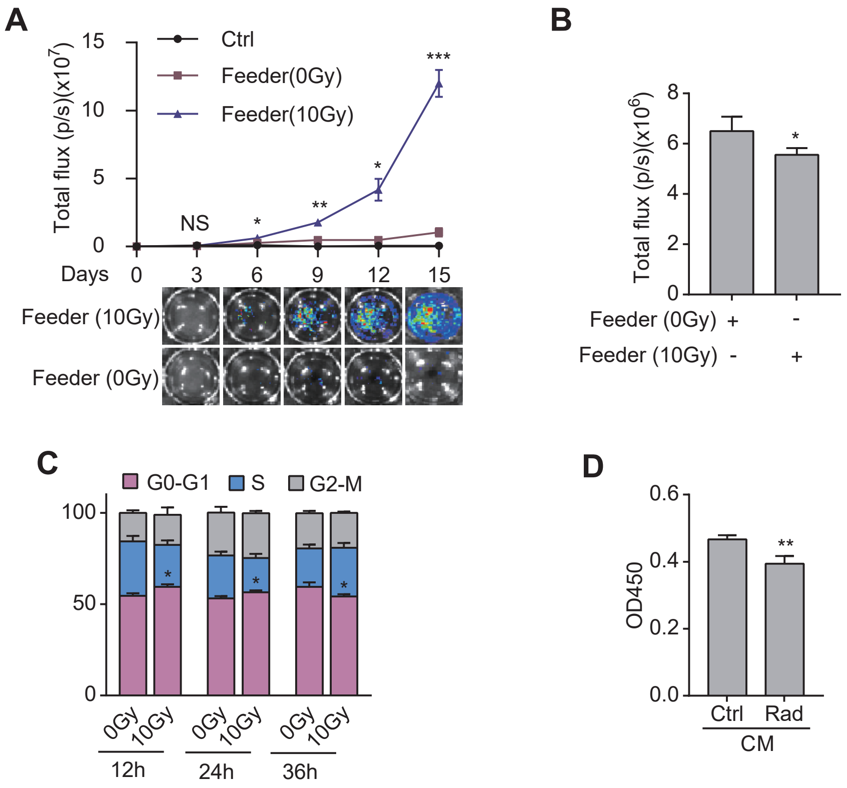

Fig. S1 Irradiated dying tumor cells dynamically regulate proliferation of reporter cells. **a** Cell proliferation curve (top) and representative bioluminescence images (down) of SW1990 reporter cells. Reporter cells were cultured alone or cocultured with unirradiated or 10Gy irradiated feeder cells. **b** Bioluminescence intensity of SW1990 reporter cells cocultured with unirradiated or 10Gy irradiated feeder cells. **c** Cell cycle distribution of SW1990 reporter cell cocultured with unirradiated or 10Gy irradiated cells. **d** Cell viability assay of cells treated with CM of unirradiated or 10Gy irradiated SW1990 cells. Data are presented as mean with SD of at least three independent experiments; *p < 0.05; **p < 0.01; ***p < 0.001 from unpaired Student's t test.
