# Supplementary material for: Dying tumor cell-derived exosomal miR-194-5p potentiates survival and repopulation of tumor repopulating cells upon radiotherapy in pancreatic cancer
Source: Mol Cancer. 2020 Mar 30;19:68. doi: 10.1186/s12943-020-01178-6 (PMC7104536; doi:10.1186/s12943-020-01178-6)
Supplement: Supplementary file 2 — Additional file 2:Figure S2. Increased secretion of exosomes from dying tumor cells inhibit cell proliferation but potentiate tumor repopulation. [file 12943_2020_1178_MOESM2_ESM.pdf]

# Supplementary Figure S2

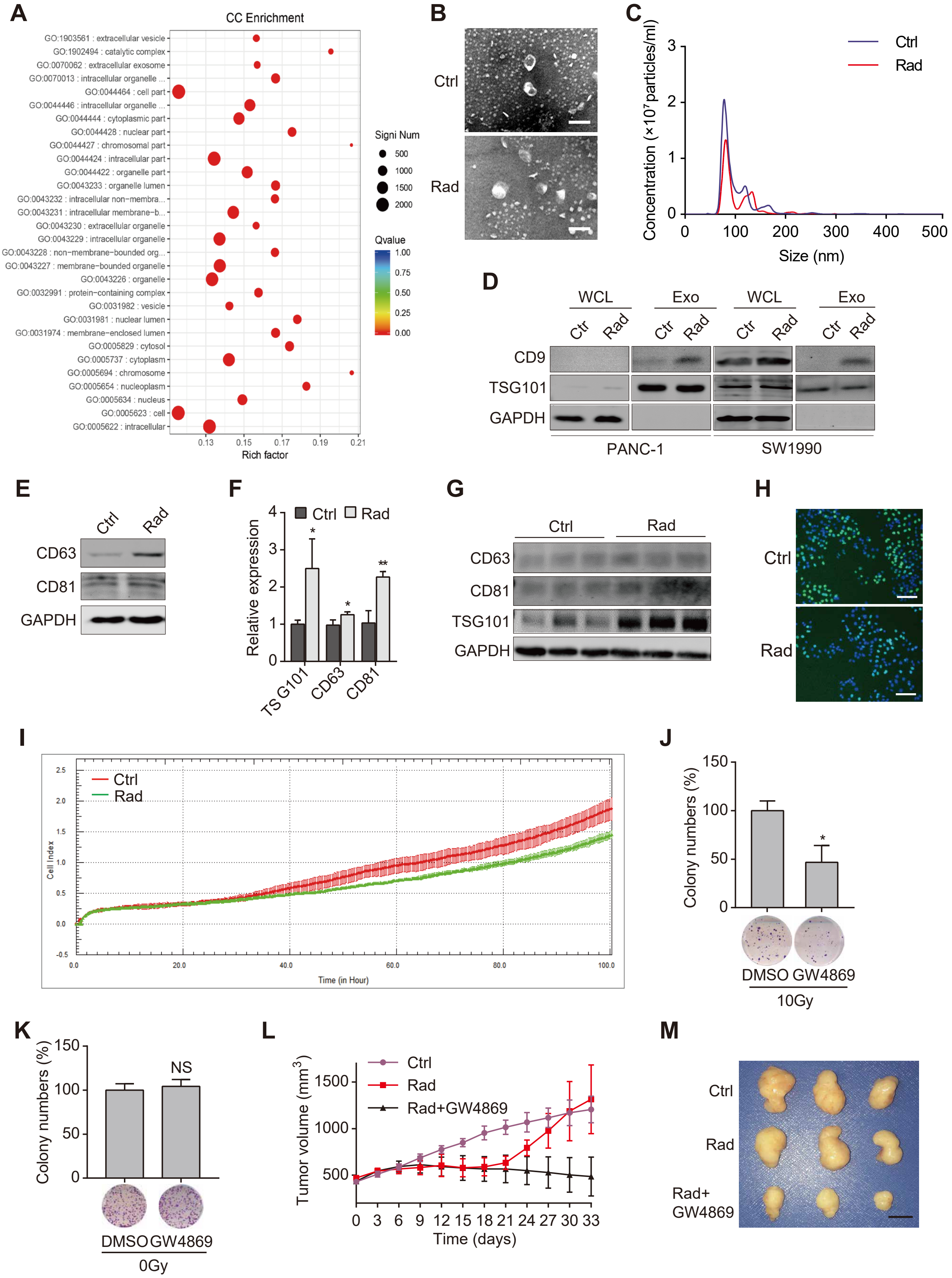

**Fig. S2** Increased secretion of exosomes from dying tumor cells inhibit cell proliferation but potentiate tumor repopulation. **a** Gene Ontology enrichment analysis of significantly changed mRNAs in PANC-1 cells after 10Gy radiation. **b** Representative transmission electron microscopy images of exosomes derived from unirradiated or 10Gy irradiated pancreatic cancer cells. Scale bar: 100 nm. **c** Representative results of nanoparticle tracking analysis detection of exosomes. **d** Western blot detecting the expression of CD9 and TSG101 in the whole cell lysates (WCL) and exosomes (exo) isolated from unirradiated or 10Gy irradiated SW1990 and PANC-1 cells. **e** Western blot detecting the expression of CD63 and CD81 in unirradiated or 10Gy irradiated SW1990 cells. **f-g** Expression of TSG101, CD63, and CD81 in unirradiated or 10Gy irradiated PDX tumor tissues detected by qPCR assay (f) and western blot analysis (g), respectively. n=3 for each group. **h** Representative images of EdU cell proliferation assay in SW1990 cells treated with exosomes derived from unirradiated or 10Gy irradiated cells. The percentage of EdU+ cells was shown in Fig. 1d. Scale bar: 100 μm. **i** Real-time cell analysis of proliferation in SW1990 cells treated with exosomes isolated from unirradiated or 10Gy irradiated cells. **j-k** Relative colony numbers (top) and representative images (down) of SW1990 cells treated with DMSO or GW4869, and subjected to 10Gy (j) or 0Gy (k) radiation. **l** Tumor growth curve of PDX tumors. The tumors were treated with 10Gy radiation with or without GW4869. This PDX was derived from another patient other than that in Figure 1H-I. n=3 for each group. **m** Tumor images from above PDX tumor-bearing mice at day 33. Scale bar: 1 cm. Data are presented as mean with SD of at least three independent experiments; *$p < 0.05$; **$p < 0.01$; NS, not significant from unpaired Student's t test.
