# Supplementary material for: Dying tumor cell-derived exosomal miR-194-5p potentiates survival and repopulation of tumor repopulating cells upon radiotherapy in pancreatic cancer
Source: Mol Cancer. 2020 Mar 30;19:68. doi: 10.1186/s12943-020-01178-6 (PMC7104536; doi:10.1186/s12943-020-01178-6)
Supplement: Supplementary file 3 — Additional file 3:Figure S3. ALDH1A1+ cells are a subpopulation of TRCs with cancer stem cell-like properties. [file 12943_2020_1178_MOESM3_ESM.pdf]

Supplementary Figure S3

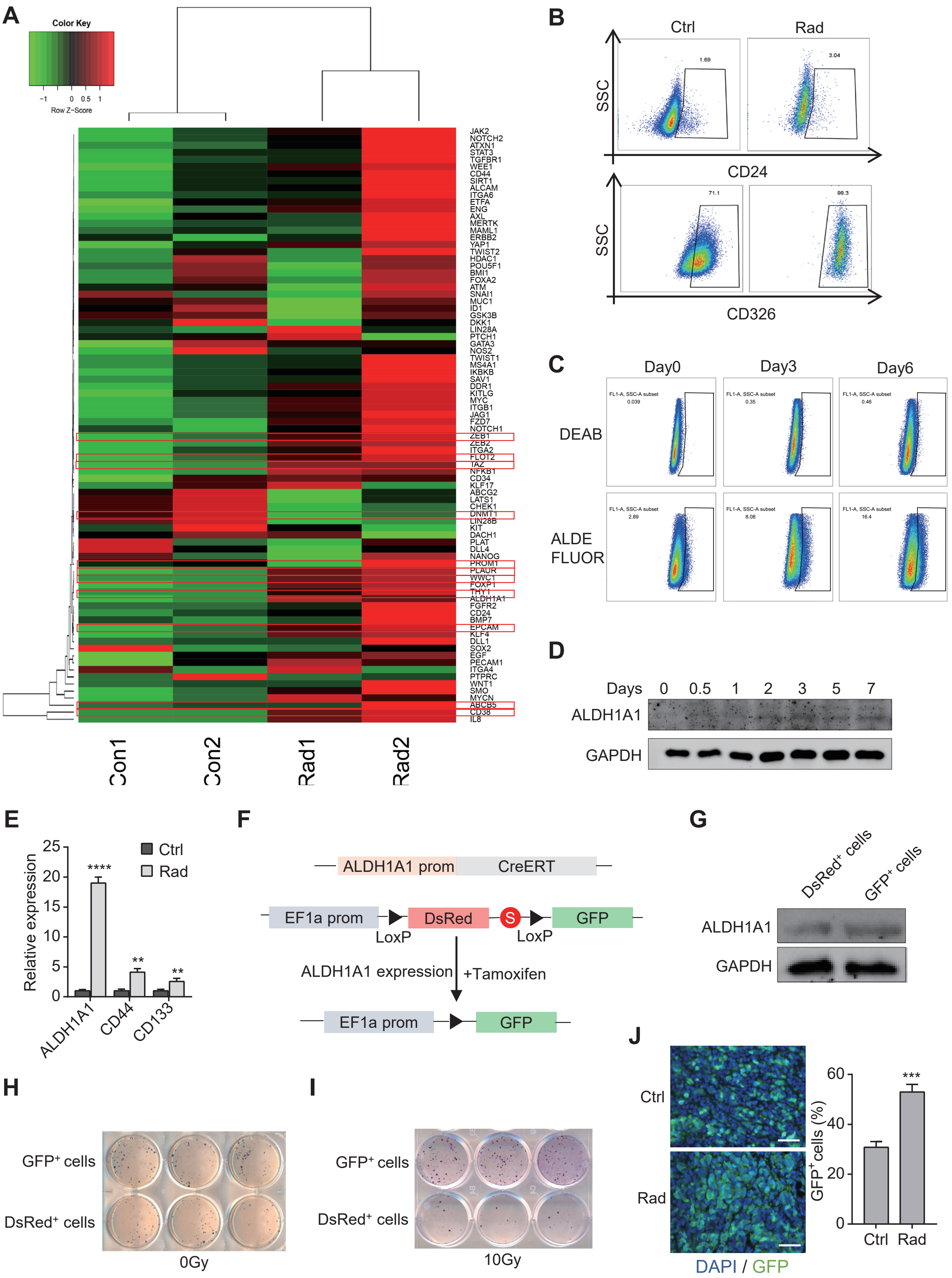

Fig. S3 ALDH1A1$^+$ cells are a subpopulation of TRCs with cancer stem cell-like properties. **a** Heatmap showing the expression of cancer stem cell-related genes by qPCR array in 10Gy irradiated or unirradiated PANC-1 cells. Experiments were performed in duplicate. The significantly changed genes were enclosed and further depicted in Fig. 2a. **b** Representative results of FACS detecting the expression of CD24 and CD326 in PANC-1 cells before and after 10Gy radiation. **c** Representative results of ALDEFLOUR™ assay in PANC-1 cells before and after 10Gy radiation. DEAB inhibited the activity of ALDH enzymes and thus served as the negative control. **d** Western blot analysis of ALDH1A1 expression in PANC-1 cells before and after 10Gy radiation. **e** qPCR assay of the relative expression of ALDH1A1, CD44 and CD133 in PDX tumor tissues that were 10Gy irradiated or unirradiated. **f** Schematic diagram of cancer cell lineage tracing system. All the tracing cells expressed DsRed without 4-OHT induction. After being treated with 4-OHT, the cells expressed ALDH1A1 would change to express GFP. **g** ALDH1A1 expression in SW1990 tracing cells. Cells were treated with 4-OHT and then DsRed$^+$ and GFP$^+$ cells were sorted by FACS. Isolated cells were then subjected to western blot analysis. **h** Soft-agar colony formation assay of sorted SW1990 tracing cells. Colonies were stained by NBT. **i** Colony formation assay of sorted SW1990 tracing cells after 10Gy radiation. **j** GFP$^+$ cells in subcutaneous tumors generated from SW1990 tracing cells. Tumors were induced by tamoxifen and subjected to radiation or not. Immunofluorescence staining of GFP was performed and the representative images (left) and quantification (right) were shown. Data are presented as mean with SD of at least three independent experiments; $^{**}p < 0.01$; $^{***}p < 0.001$; $^{****}p < 0.0001$ from unpaired Student's t test.
