# Supplementary material for: Dying tumor cell-derived exosomal miR-194-5p potentiates survival and repopulation of tumor repopulating cells upon radiotherapy in pancreatic cancer
Source: Mol Cancer. 2020 Mar 30;19:68. doi: 10.1186/s12943-020-01178-6 (PMC7104536; doi:10.1186/s12943-020-01178-6)
Supplement: Supplementary file 4 — Additional file 4:Figure S4. miR-194-5p suppresses pancreatic cancer cell proliferation, migration and invasion, but promotes DNA damage repair. [file 12943_2020_1178_MOESM4_ESM.pdf]

Supplementary Figure S4

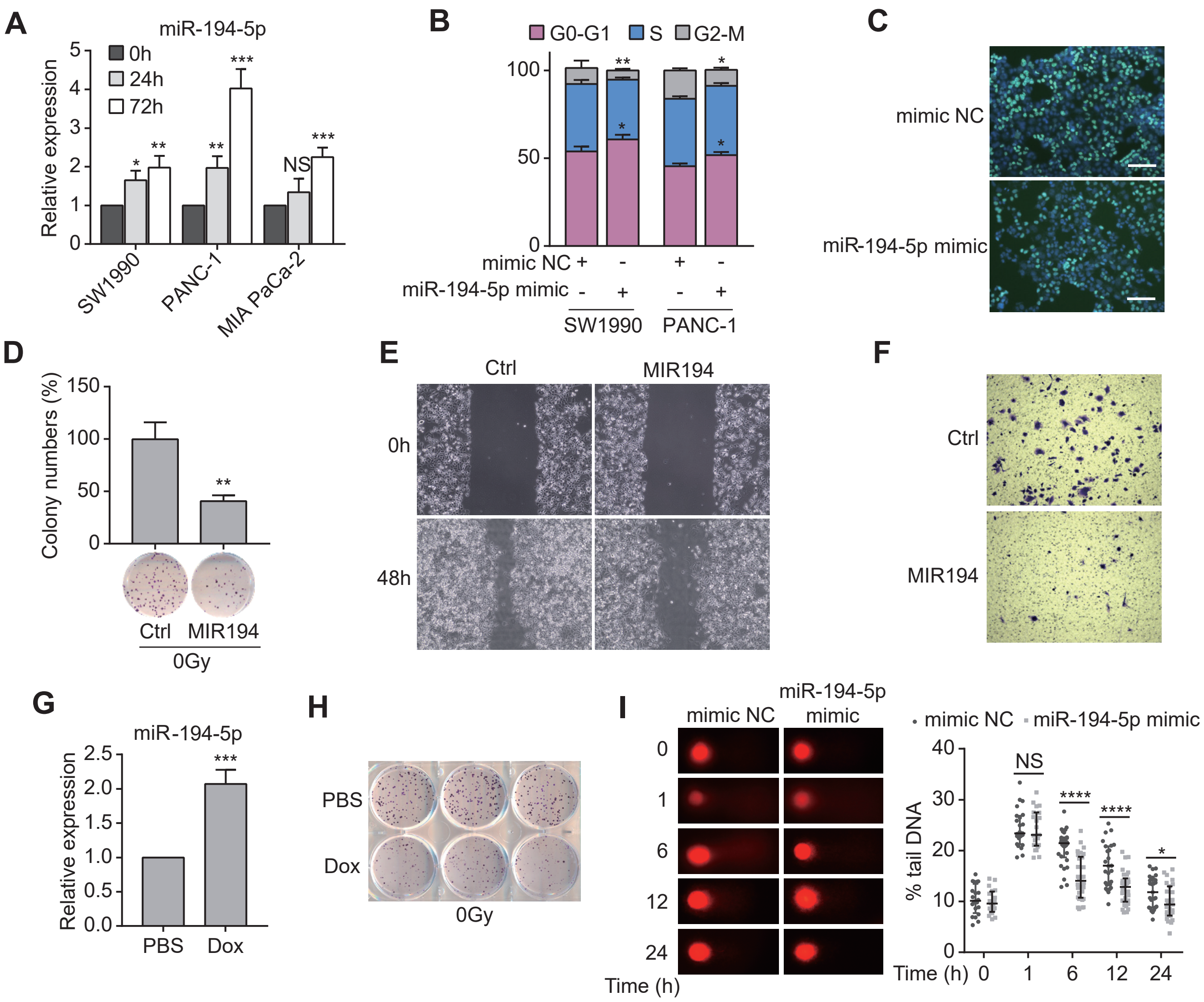

Fig. S4 miR-194-5p suppresses pancreatic cancer cell proliferation, migration and invasion, but promotes DNA damage repair. **a** Relative expression of miR-194-5p in pancreatic cancer cells before and after 10Gy radiation by qPCR assay. **b** Cell cycle distribution of pancreatic cancer cells treated with miR-194-5p mimics or mimic NC. **c** Representative images of EdU staining in SW1990 cells treated with miR-194-5p mimics or mimic NC. Scale bar: 100 μm. **d** Relative colony numbers (top) and representative images (down) of SW1990 cells that stably overexpress miR-194-5p or control. **e** Wound healing assay of PANC-1 cells that stably overexpress miR-194-5p or control. **f** Transwell assay (8 μm pole) of PANC-1 cells that stably overexpress miR-194-5p or control. **g** Relative expression of miR-194-5p in SW1990 tet-on cells. Cells were treated with doxycycline (Dox) (1μg/ml) or PBS. **h** Colony formation assay of SW1990 tet-on cells treated with doxycycline or PBS. **i** Representative images (left) and quantifications (right) of comet assay in PANC-1 cells transfected with miR-194-5p mimics or mimic NC and subjected to 2Gy radiation. Data are presented as mean with SD of at least three independent experiments; *$p < 0.05$; **$p < 0.01$; ***$p < 0.001$; ****$p < 0.0001$; NS, not significant from unpaired Student's t test.
