# Supplementary material for: Dying tumor cell-derived exosomal miR-194-5p potentiates survival and repopulation of tumor repopulating cells upon radiotherapy in pancreatic cancer
Source: Mol Cancer. 2020 Mar 30;19:68. doi: 10.1186/s12943-020-01178-6 (PMC7104536; doi:10.1186/s12943-020-01178-6)
Supplement: Supplementary file 5 — Additional file 5:Figure S5. E2F3 is a target of miR-194 and promotes pancreatic cell proliferation, inhibits DNA damage repair. [file 12943_2020_1178_MOESM5_ESM.pdf]

Supplementary Figure S5

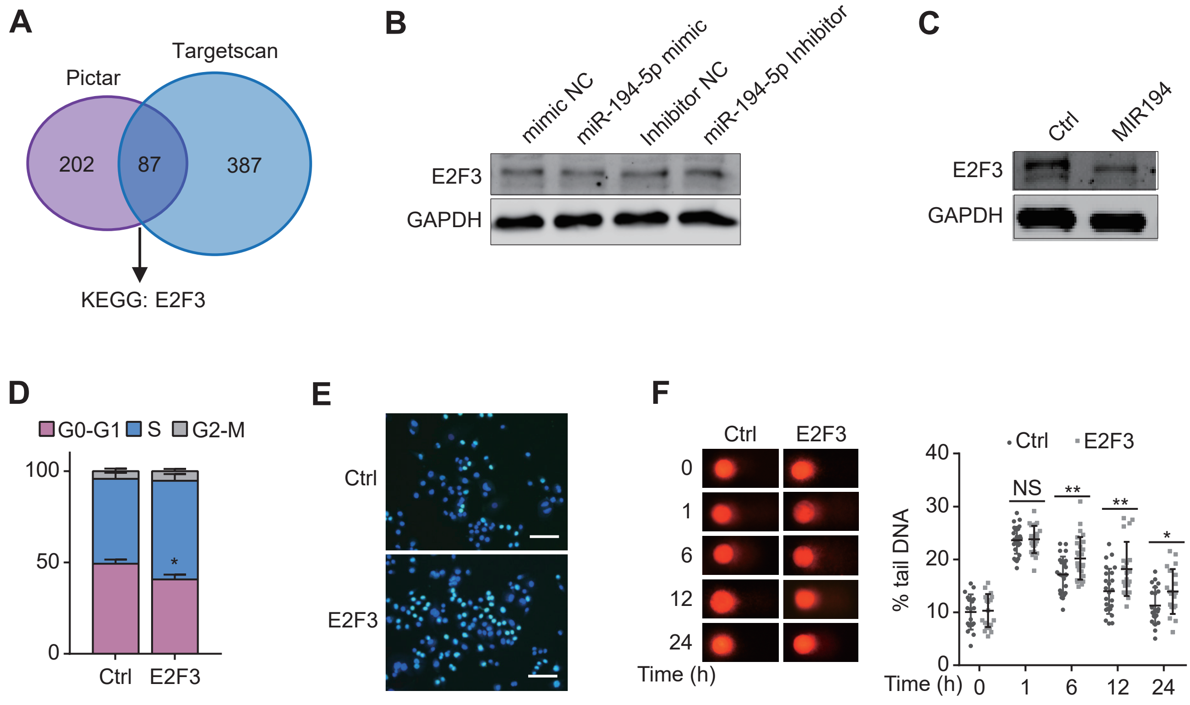

Fig. S5 E2F3 is a target of miR-194-5p and promotes pancreatic cell proliferation, inhibits DNA damage repair. **a** Venn diagram of the predicted targeting mRNAs of miR-194-5p by Pictar and Targetscan. KEGG annotation identified E2F3 as the only gene that is associated with cell cycle regulation among the overlapped genes of prediction. **b** Western blot results of E2F3 expression in SW1990 cells transfected with miR-194-5p mimics or inhibitor and their corresponding negative controls. **c** Western blot results of E2F3 expression in SW1990 cells that stably overexpress E2F3 or control. **d-e** Cell cycle distribution (d) and representative images of EdU staining (e) of SW1990 cells that stably overexpress E2F3 or control. Scale bar: 100 µm. **f** Representative images (left) and quantifications (right) of comet assay in PANC-1 cells that stably overexpress E2F3 or control and subjected to 2Gy radiation. Data are presented as mean with SD of at least three independent experiments; *$p < 0.05$; **$p < 0.01$; NS, not significant from unpaired Student's t test.
