# Supplementary material for: Dying tumor cell-derived exosomal miR-194-5p potentiates survival and repopulation of tumor repopulating cells upon radiotherapy in pancreatic cancer
Source: Mol Cancer. 2020 Mar 30;19:68. doi: 10.1186/s12943-020-01178-6 (PMC7104536; doi:10.1186/s12943-020-01178-6)
Supplement: Supplementary file 6 — Additional file 6:Figure S6. HMGA2 promotes pancreatic cancer stemness and progression, but inhibits DNA damage repair. [file 12943_2020_1178_MOESM6_ESM.pdf]

Supplementary Figure S6

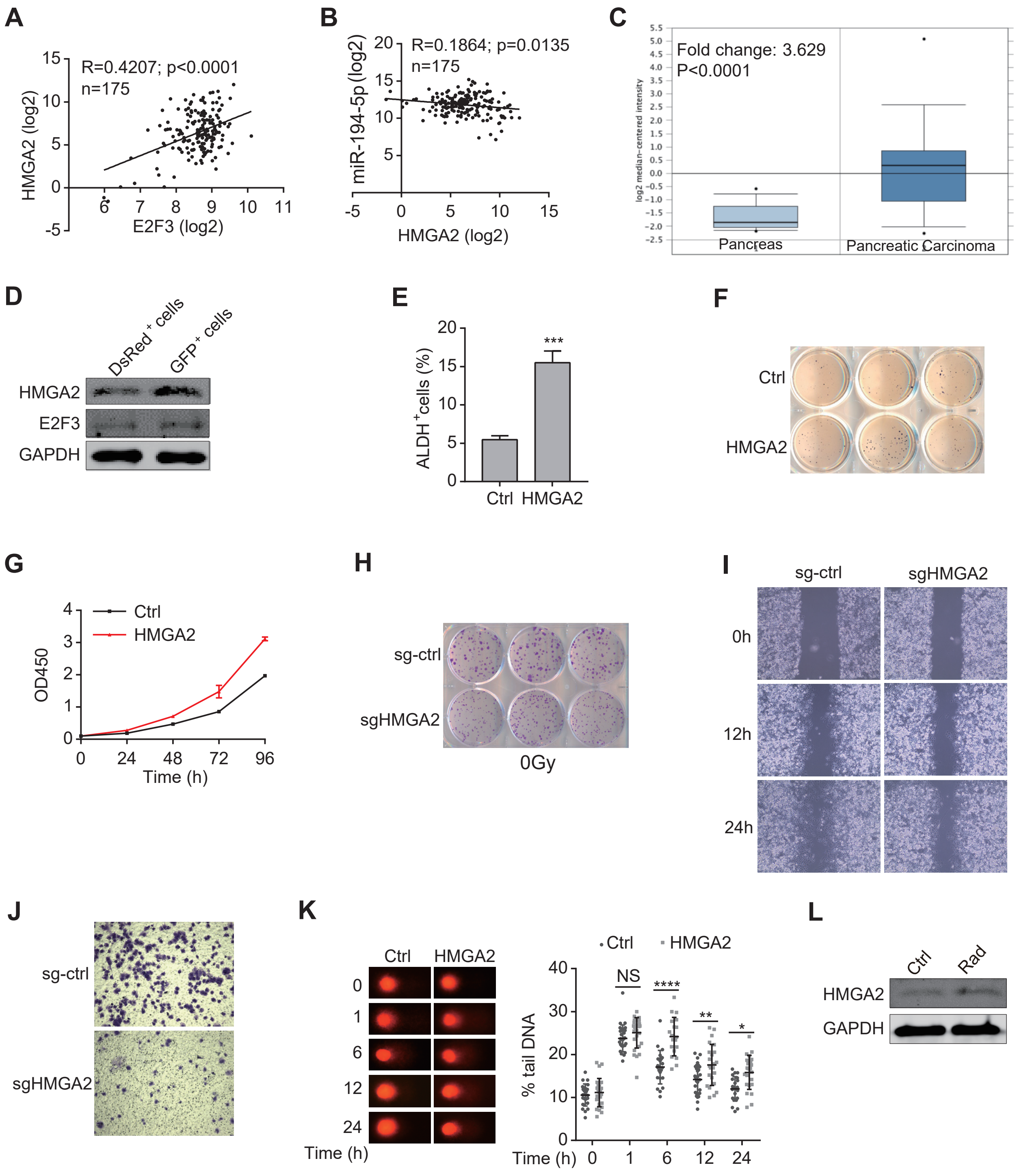

Fig. S6 HMGA2 promotes pancreatic cancer stemness and progression, but inhibits DNA damage repair. **a** Scatter diagram and linear regression of E2F3 expression (log2) and HMGA2 expression(log2) in pancreatic cancer tissues. Data were obtained from the TCGA database and F Test was used to determine the significance of correlation. **b** Scatter diagram and linear regression of HMGA2 expression (log2) and miR-194-5p expression (log2) in pancreatic cancer tissues. Data were obtained from the TCGA database and F Test was used to determine the significance of correlation. **c** Expression of HMGA2 in normal pancreas tissues and pancreatic carcinomas. Data were obtained from the Oncomine database. 36 pancreatic carcinomas and 16 paired normal samples were analyzed with Human Genome U133 Plus 2.0 Array. p value was calculated by t-Test. **d** Expression of HMGA2 and E2F3 in the sorted tracing SW1990 cells. **e** Percentage of $ALDH^+$ cells in SW1990 cells that overexpress HMGA2 or negative control. Cells were analyzed using ALDEFLOUR™ kit. **f-g** Soft-agar colony formation (f) and proliferation curve (g) of SW1990 cells that stably overexpress HMGA2 or negative control. The colonies were stained by NBT. **h-j** Representative images of plate colony formation assay (h), wound healing assay (i) and transwell assay (j) of the indicated PANC-1 cells. **k** Representative images (left) and quantifications (right) of comet assay in SW1990 cells that overexpress HMGA2 or negative control and subjected to 2Gy radiation. **l** Western blot results of HMGA2 expression in unirradiated or 10Gy irradiated GFP^+ cells . Data are presented as mean with SD of at least three independent experiments; $*p < 0.05$, $**p<0.01$, $***p < 0.001$, $****p<0.0001$, NS, not significant from unpaired Student's t test.
