# Supplementary material for: Dying tumor cell-derived exosomal miR-194-5p potentiates survival and repopulation of tumor repopulating cells upon radiotherapy in pancreatic cancer
Source: Mol Cancer. 2020 Mar 30;19:68. doi: 10.1186/s12943-020-01178-6 (PMC7104536; doi:10.1186/s12943-020-01178-6)
Supplement: Supplementary file 7 — Additional file 7:Figure S7. Expression of PTGS2 and exosome-related proteins in PANC-1 cells after radiation. [file 12943_2020_1178_MOESM7_ESM.pdf]

Supplementary Figure S7

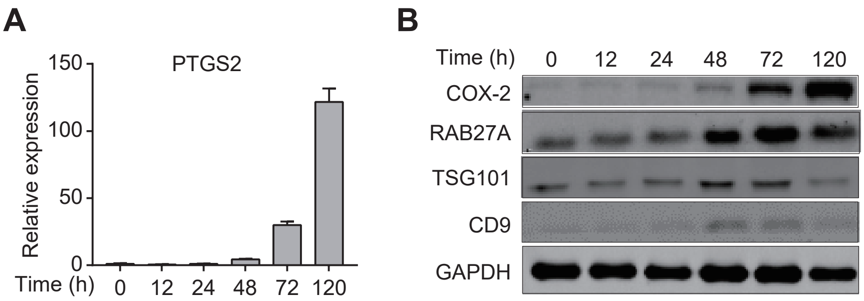

Fig. S7 Expression of PTGS2 and exosome-related proteins in PANC-1 cells after radiation. **a** qPCR quantification of PTGS2 expression in PANC-1 cells before and after 10Gy radiation. Data are presented as means with SD. **b** Western blot results of COX-2, RAB27A, TSG101 and CD9 expression in PANC-1 cells before and after 10Gy radiation.
