# Supplementary material for: Dying tumor cell-derived exosomal miR-194-5p potentiates survival and repopulation of tumor repopulating cells upon radiotherapy in pancreatic cancer
Source: Mol Cancer. 2020 Mar 30;19:68. doi: 10.1186/s12943-020-01178-6 (PMC7104536; doi:10.1186/s12943-020-01178-6)
Supplement: Supplementary file 8 — Additional file 8:Fig. S8. Effects and mechanisms of aspirin in suppressing pancreatic cancer repopulation. [file 12943_2020_1178_MOESM8_ESM.pdf]

Supplementary Figure S8

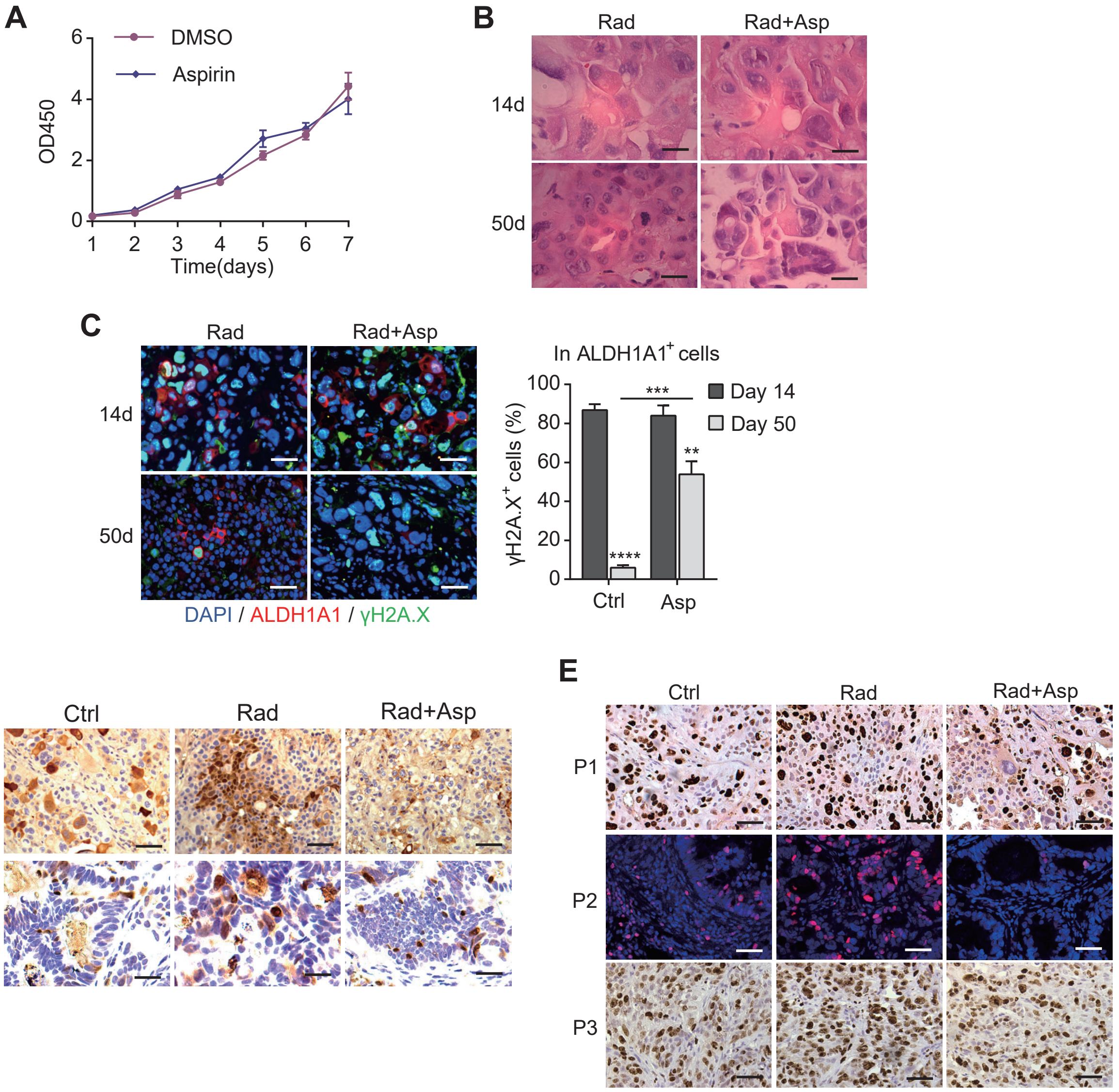

Fig. S8 Effects and mechanisms of aspirin in suppressing pancreatic cancer repopulation. **a** Proliferation curve of aspirin or DMSO-treated SW1990 cells detected by CCK-8 assay. **b** Representative images of H&E staining of the PDX tissues treated with 10Gy radiation with or without aspirin. Scale bar: 20 µm. **c** Representative images (left) and quantifications (right) of immunofluorescence staining of ALDH1A1 (red) and γH2A.X (green) in PDX tumor tissues treated as in Figure S8B. Scale bar: 50 µm. **d-e** Representative images of IHC and immunofluorescence staining of ALDH1A1 (d) and Ki-67 (e) in PDX tumor tissues treated as in Figure S8B. Scale bar: 50 µm. Data are represented as mean with SD; $p < 0.01$; ***$p < 0.001$; ****$p < 0.001$ from unpaired Student's t test.
