# Supplementary material for: Dying tumor cell-derived exosomal miR-194-5p potentiates survival and repopulation of tumor repopulating cells upon radiotherapy in pancreatic cancer
Source: Mol Cancer. 2020 Mar 30;19:68. doi: 10.1186/s12943-020-01178-6 (PMC7104536; doi:10.1186/s12943-020-01178-6)
Supplement: Supplementary file 9 — Additional file 9:Fig. S9. Schematic diagram of the plasmid constructs. [file 12943_2020_1178_MOESM9_ESM.pdf]

Supplementary Figure S9

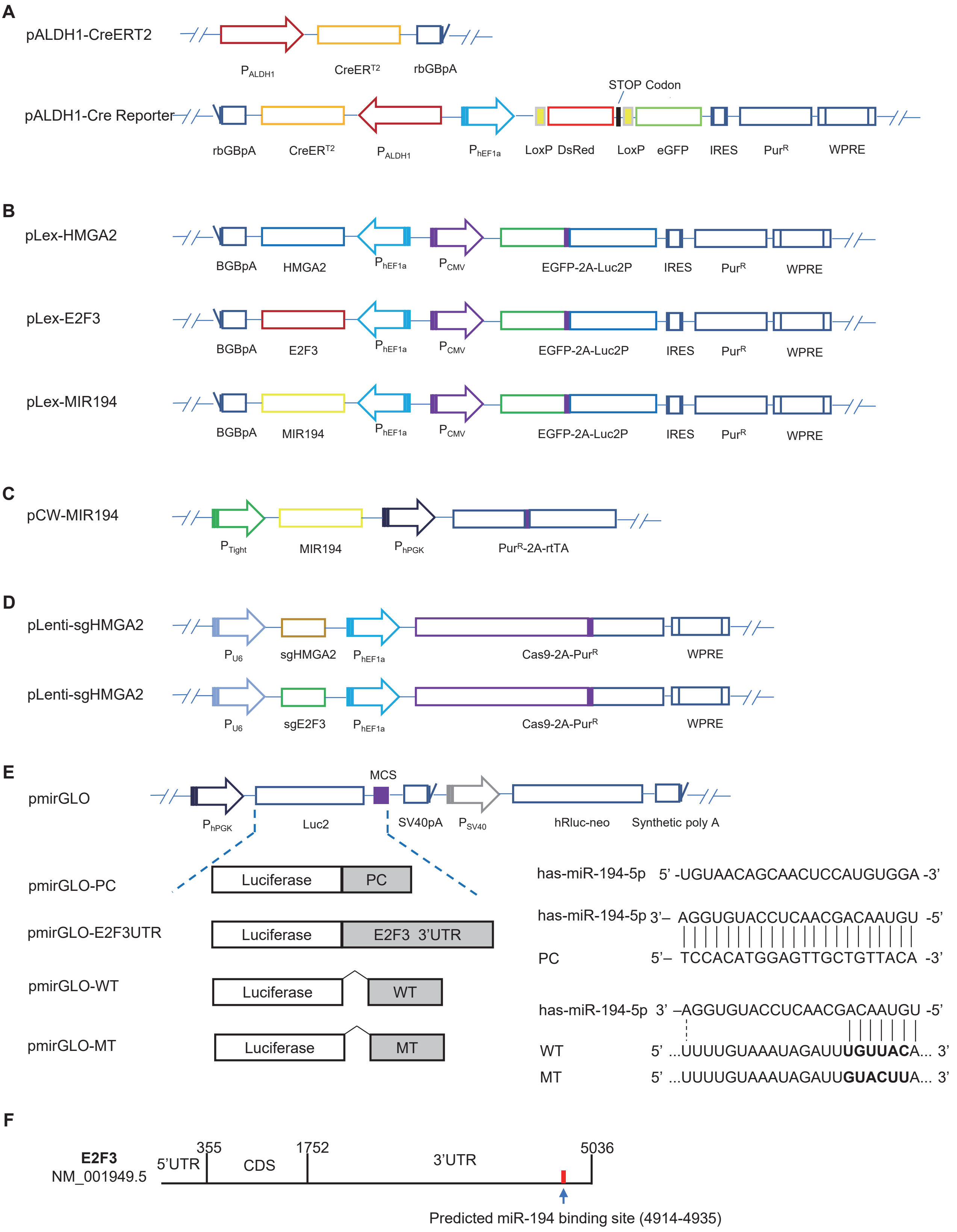

Fig. S9 Schematic diagram of the plasmid constructs. **a** Schematic diagram of the lineage tracing plasmid. ALDH1A1 promoter was amplified to replace the CAG promoter in plasmid pCAG-CreERT2 to construct the plasmid pALDH1-CreERT2. The ALDH1-CreERT2 cassette was then inserted into plasmid Cre reporter to construct the lineage tracing plasmid pALDH1-Cre Reporter. Principle of the plasmid was depicted in Figure S3F. **b** Lentiviral expression vector for overexpression of target genes. The CDS region of the HMGA2, E2F3 and the gene with flanking sequence of MIR194-1 was cloned into the lentiviral expression vector pLex-MCS to generate pLex-HMGA2, pLex-E2F3 and pLex-MIR194. **c** Lentiviral expression vector for the tetracycline-dependent expression (tet-on) of miR-194-5p. The gene with flanking sequence of MIR194-1 was cloned into plasmid pCW-Cas9. The plasmid contained the elements for the tetracycline-dependent gene expression, including Tight TRE promoter and improved tetracycline-controlled transactivator rtTA-Advance. **d** Lentiviral expression vector for HMGA2 or E2F3 knock-out based on CRISPR/Cas9 system. Targeting sequence of sgRNA for HMGA2 or E2F3 was cloned into the plasmid Lenti-CRISPR v2. This plasmid contained the elements required for knocking out HMGA2 or E2F3, including Cas9 enzyme and sgRNA scaffold. **e** Schematic diagram of plasmids for identifying the targeting site of miR-194 in E2F3. PC, positive control which were perfectly complementary to miR-194-5p; UTR, full length of the 3'UTR of E2F3; WT, predicted targeting sequence of miR-194-5p binding site; MT, predicted targeting sequence of miR-194-5p binding site with mutations in the seed sequence. The PC, E2F3UTR, WT and MT sequencing was cloned into the MCS site downstream of Luc2 (lower left). miR-194-5p and its complete complementary sequence, predicted miR-194 binding sequence in E2F3 and the mutated sequence were shown in the lower right. **f** Diagram of the E2F3 mRNA. There is a miR-194-binding site located in the E2F3 3'UTR.
