# Supplementary material for: Dying tumor cell-derived exosomal miR-194-5p potentiates survival and repopulation of tumor repopulating cells upon radiotherapy in pancreatic cancer
Source: Mol Cancer. 2020 Mar 30;19:68. doi: 10.1186/s12943-020-01178-6 (PMC7104536; doi:10.1186/s12943-020-01178-6)
Supplement: Supplementary file 10 — Additional file 10. Supplementary materials and methods. [file 12943_2020_1178_MOESM10_ESM.docx]

**Supplementary Materials and Methods**

**Cell lines and cell culture**

Human pancreatic cancer cell line SW1990 (ATCC^®^ CRL-2172) was cultured in RPMI 1640 (Gibco) with 10% FBS (Gibco). Human pancreatic cancer cell line PANC-1 (ATCC^®^ CRL-1469), MIA PaCa-2 (ATCC^®^ CRM-CRL-142) and human kidney epithelial cell line HEK 293T (ATCC^®^ CRL-1126) were cultured in DMEM (Hyclone) with 10% FBS (Gibco). Antibiotics (100 U/ml Penicillin and 100 μg/ml Streptomycin, Gibco) was routinely added to the media. All the cells were incubated in a humidified incubator containing 5% CO_2_ at 37 ℃. All the cell lines were confirmed by STR identification and routinely tested for mycoplasma-free using MycoFree^TM^ mycoplasma detection kit (Genechem).

**Isolation and culture of primary pancreatic cancer cells**

Human pancreatic cancer tissues were obtained by surgery and washed twice with DMEM/F12 (1:1) (Hyclone) containing 5% FBS, 100 U/ml Penicillin and 100 μg/ml Streptomycin. Tumor tissues were cut into small pieces with scissors, and digested with digestion buffer (DMEM/F12 containing 10%FBS, 50 μg/ml DNA enzyme (Sigma) and 1mg/ml type IV collagen enzyme (Sigma)) for 30 min at 37 ℃ in the incubator shaker at 150 rpm. Cells were then filtered through a 70 μm nylon cell strainer (Falcon) and plated in the 6-well plate precoated with rat-tail type I collagen (Gibco) solution (50 μg/ml type I collagen in 10 mM acetic acid) for regular cell culture. Cell culture media were composed of DMEM/F12 plus recombinant 10 ng/ml epidermal growth factor (PeproTech), 30 μg/ml bovine pituitary extract (sigma), 2% FBS (Gibco), and 1% antibiotic-antimycotic (Gibco). Cancer-associated fibroblasts were removed by trypsinization 24 hrs after seeding. The primary cancer cells were then subjected to subsequent experiments.

**Tumor-bearing mouse models**

The generation of patient-derived xenograft (PDX) mouse models was performed as previous described [1, 2]. In brief, tumors were cut into small pieces and subcutaneously implanted into the right groin of 6 to 8-week-old male BALB/c nude mice. The PDX tissues of 3-10 passages were used for further experiments. The diameter of tumors was measured by digital caliper, and tumor volume was calculated by the formula, volume = 0.5 × length ×width^2^. When tumor volume reached 500 mm^3^, mice were subjected to radiation and/or intraperitoneal injection of GW4869 (Selleckchem, 2.5 μg/g body weight) every two days [3], or treated with aspirin (Sigma-Aldrich, 600 μg/ml in drinking water) [4] for 2 weeks. Diameters of the tumors were measured every 2-3 days. Mice were sacrificed when the volume of tumors reached 2 cm^3^ or the mice showed significant cachexy.

The *in vivo* lineage tracing mouse model was established in 6-8w male BALB/c nude mice with the tracing SW1990 cells. In brief, SW1990 cells were stablely transfected with the lineage tracing vectors. The stable tracing pancreatic cancer cells were then resuspended in serum-free medium at a density of 2×10^6^ cells/ml, and further injected into the right groin of mice at the volume of 100μl. When the volume of tumors reached 500 mm^3^, mice were subjected to radiation and analyzed 2 weeks later.

**Plasmid construction**

The lineage tracing plasmid was constructed by standard molecular cloning technology. In brief, the promoter of ALDH1A1 gene was amplified from genomic DNA of HEK 293T cells by regular PCR. The amplified promoter fragments were then cloned into the expression plasmid pCAG-CreERT2, which was a gift from Connie Cepko (Addgene plasmid ^#^14797) [5], by double digestion with restriction enzyme Spe I (New England Biolabs, NEB) and EcoR I (NEB), and the resulted recombinant plasmid was named pALDH1-CreERT2. Further, pALDH1-CreERT2 was applied to generate the expression cassette ALDH1-CreERT2 by a series of sequential manipulations, which was first digested with Nde I, then filled the ends with T4 DNA polymerase (NEB), and last digested with Age I (NEB). Likewise, the vector backbone was generated from plasmid Cre Reporter, a gift from Niels Geijsen (Addgene plasmid ^#^62732) [6], by the same manipulations as the expression cassette was generated. The vector backbone and the expression cassette were then ligated by T4 DNA ligase (NEB) to generate the lineage tracing plasmid pALDH1-Cre Reporter.

The lentivirus-based gene overexpression plasmids were constructed as previous reported [1]. In brief, the coding sequence (CDS) of HMGA2 (NM_003483.4) and E2F3 (NM_001949.5) were amplified from total RNA of HEK 293T cells by regular RT-PCR, while miR-194-5p expression region was amplified from genomic DNA of HEK 293T cells by regular PCR. All the products were doubly digested by Nhe I/BamH I (NEB) and cloned into pLex-MCS [1] to generate the recombinant plasmids pLex-HMGA2, pLex-E2F3, pLex-MIR194, respectively. Meanwhile, the amplified miR-194 expression region was also cloned into the doxycycline-induced expression plasmid pCW-Cas9, a gift from Eric Lander & David Sabatini (Addgene plasmid ^#^50661), both by double digestion with BamH I and Nhe I. The resulted plasmid was named pCW-MIR194, in which miR-194 expression was under the doxycycline induction.

The sgRNAs for CRISPR/Cas9-mediated *HMGA2* gene (Gene ID: 8091) and *E2F3* gene (Gene ID: 1871) knockout were designed by the online tool (https://portals.broadinstitute.org/gpp/public/analysis-tools/sgrna-design) [7, 8]. The synthesized sgRNAs were cloned into the plasmid LentiCRISPR v2, a gift from Dr. F Zhang (Addgene plasmid ^#^52961) [9], by the Golden Gate Assembly following the protocol provided by Dr. F Zhang, and the resulted recombinant plasmids were named pLenti-sgHMGA2, pLenti-sgE2F3.

All the plasmids for identification of the miR-194 targeting sites in E2F3 by the dual-luciferase reporter assay were based on plasmid pmirGLO (Promega). Briefly, the 3’UTR region of E2F3 was amplified from total RNA of HEK 293T cells by RT-PCR, and cloned into pmirGLO by double digestion with Nhe I/Sal I to generate the plasmid pmirGLO-E2F3UTR. Furthermore, the complete complement sequence of miR-194 (as positive controls, PC) and the corresponding putative miR-194 target site (WT) as well as the mutant sequence (MT) in E2F3 were synthetized, annealed and then cloned into the Nhe I/Sal I double digested pmirGLO to generate the reporter plasmid pmirGLO-PC, pmirGLO-WT and pmirGLO-MT, respectively.

All the primers and oligos were synthetized by Sangon Biotech (Shanghai, China), and their sequences were shown in Additional file (Additional file 11: Table S1). All the PCR fragments were verified by sequencing (Sangon Biotech). All the constructed plasmids were schematically represented in Additional file (Additional file 9: Figure S9).

**Lentivirus production and infection**

Lentivirus production was carried out as previously described [1]. In brief, HEK 293T cells were passaged 1 day before transfection. The recombinant lentiviral vectors were co-transfected with the packaging plasmids pSPAX2 and pMD2.G using Lipofectamine™ 3000 Transfection Reagent (Invitrogen) according to the manufacturer’s instructions. The supernatants were collected at 48, 72 hrs later, respectively, and centrifuged at 10,000g for 30 min to remove cell debris. Lentivirus was then collected by ultracentrifuging (Beckman Coulter) at 80,000g for 90 min. All the purified lentiviruses were stocked at -80 ℃ for further experiments.

Lentivirus infection of pancreatic cancer cells was performed in the presence of polybrene (Sigma-Aldrich, 8 μg/ml) for 12 hrs. The infected positive cells were then selected by puromycin (Selleckchem,1μg/ml) selection or FACS with MoFlo High-Performance Cell sorter (Beckman Coulter).

**CRISPR/Cas9-mediated gene knockout**

Cells at the 6-well plate were infected with the indicated lentivirus and selected by puromycin for 5 days. Then, these selected cells were sorted by FACS and seeded into the 96-well plate at a density of 1 cell/well. The single cell clone was cultured and amplified, and tested for the expression of targeted proteins by immunoblotting. The clones that were absent from the expression of targeted protein were selected, and the targeted regions were accordingly amplified by PCR for sanger sequencing (Sangon Biotech) and determination of the mutations. The verified clones were then mixed for subsequent experiments.

**Isolation and analysis of exosomes**

To isolate exosomes in cell supernatants, cells were cultured in the media with exosomes-depleted FBS (ultracentrifuged at 12,000g for 18 hrs at 4 ℃). Immediate before irradiation, the fresh culture media containing 2% exosome-depleted FBS were added. Cell culture media were collected 24 hrs after irradiation and subjected to differential centrifugation for isolation of exosomes as previous described [10], including sequential centrifugation of 300g, 2,000g and 10,000g for removal of live and dead cells and cell debris, and ultracentrifugation of 100,000g twice for removal of contaminated proteins. The pellets were then used for further analysis. For RNA analysis, TRIzol Reagent (Invitrogen) was directly added to the pellets for RNA extraction. For protein analysis, 1×SDS loading buffer (Beyotime Biotechnology, Shanghai) was added to the pellets and heated at 100 ℃ for 5 min and then subjected to immunoblotting. For NTA analysis, exosomes were resuspended in PBS and diluted to proper concentrations for detection by NanoSight NS300 (Malvern Panalytical). For TEM analysis, exosomes were also resuspended in PBS and subjected to negative staining with phosphotungstic acid for TEM imaging and analysis (Philips, CM120). For functional assays, the resuspended exosomes were added to the media of cancer cells.

To isolate exosomes in plasma, plasma was diluted with PBS in the ratio of 1:10 and sequentially centrifuged at 2000g for 10 min and 10,000g for 30 min to remove cell debris. The following ultracentrifugation and further analysis were performed as above.

**RNA extraction and quantification**

The TRIzol™ reagent (Invitrogen) was used to extract the total RNAs of isolated exosomes, cells and pancreatic cancer tissues. PrimeScript™ RT reagent Kit (Takara) was used for reverse transcription of RNA. TB Green^TM^ Premix Ex Taq^TM^ (Takara) was used for qPCR, which was performed in QuantStudio 6 Flex (Applied Biosystems). All above experimental operations followed the manufacturer’s instructions. All the related oligos and primers were synthetized by Sangon Biotech (Shanghai, China), and the sequences were listed in Additional file (Additional file 11: Table S1). The relative gene expression was calculated by the 2^-ΔΔCt^ algorithm.

**qPCR array**

The Human Cancer Stem Cells RT² Profiler PCR Array (Cat. No. 330231 PAHS-176ZA, Qiagen) was used to profile the expression of cancer stem cell-related genes in the residual surviving pancreatic cancer cells after radiation. In brief, the robust PANC-1 cells were exposed to 10Gy irradiation, and further cultured for 9 days to harvest the residual surviving cells. Total RNAs of the surviving cells were extracted with TRIzol™ Reagent (Invitrogen), and subjected to reverse transcription with RT^2^ First Strand Kit. The cDNAs were then mixed with an appropriate RT^2^ SYBR Green Mastermix, which were further aliquoted into the wells of the RT^2^ Profiler PCR Array. Quantitative PCR was then performed in QuantStudio 7 Flex (Applied Biosystems). All above experiments were routinely carried out. The relative expression of the related genes was determined using data from the real-time cycler and the ΔΔCt method.

**RNA sequencing**

High throughput sequencing of exosomal miRNAs was performed by CloudSeq Biotech (Shanghai, China). In short, the quantity and purity of total exosomal RNAs were determined by using a NanoDrop ND-100 (Thermo Fisher). Total RNAs of samples were used to prepare the miRNA sequencing libraries, and ~150 bp PCR amplicons (corresponding to ~22nt miRNAs) were selected. The libraries were then applied for RNA sequencing with Illumina HiSeq sequencer (Illumina) by routine.

RNA-Seq of irradiated pancreatic cancer cells of SW1990 and PANC-1 was performed by Sangon Biotech (Shanghai, China). Briefly, the quality and quantity of cellular RNAs were assessed by a NanoPhotometer^®^ spectrophotometer (IMPLEN) and an Agilent 2100 Bioanalyzer (Agilent Technologies). A total amount of 2 μg RNA per sample was used for construction of the sequencing libraries with VAHTSTM mRNA-seq V2 Library Prep Kit for Illumina^®^ following the manufacturer’s recommendations, and index codes were added to attribute sequences to each sample. The libraries were then quantified and pooled. Paired-end sequencing of the library was performed on the HiSeq XTen sequencers (Illumina).

**Western blot assay**

Cells and pancreatic cancer tissues were collected as above, and subjected to lysis with RIPA buffer (Thermal Fisher) plus the complete protease inhibitor cocktail (Roche) for 10 min on ice. The lysates were then centrifugated at 15,000g for 30 min at 4 ℃ to remove cell debris. Protein concentrations were determined by BCA Protein Assay Kit (Beyotime Biotechnology, Shanghai). Proteins were then separated by regular SDS-PAGE electrophoresis and transferred to nitrocellulose membrane. Membranes were blocked with 5% milk in TBS and then incubated with the primary antibody, including anti-GAPDH (CST, 2118), anti-ALDH1A1 (CST, 54135), anti-COX-2 (CST, 12282), anti-TSG101 (Abcam, ab83), anti-CD63 (Santa Cruz Biotechnology, sc-5275), anti-CD81 (Santa Cruz Biotechnology, sc-7637), anti-Phospho-Histone H2A.X (Ser139) (CST, 2577), anti-CD9 (CST, 13403), anti-RAB27A (CST, 69295), anti-Phospho-ATM (Ser1981) (CST, 13050), anti-E2F3 (Proteintech, 27615-1-AP), and anti-HMGA2 (CST, 8179). All antibodies were used as recommended by the manufacturer. After primary antibody incubation, the secondary antibodies IRDye^®^ 800CW Goat anti-Rabbit IgG and IRDye^®^ 680RD Goat anti-Mouse IgG (LI-CDR) were added and the membranes were then subjected to image by Odyssey® Imaging Systems (LI-CDR).

**Dual luciferase reporter assay**

The Dual-Luciferase^®^ Reporter (DLR™) Assay System (Promega) was applied to verify the target sites of miR-194 in E2F3. Briefly, PANC-1 cells were co-transfected with pmirGLO plasmids containing miR-194 target sites or 3’UTR of E2F3 as previous described and miR-194 mimics or mimic NC by using the SE Cell Line 4D-Nucleofector^®^ X Kit L (Lonza) according to the manufacturer’s protocol. Cells were collected 48 hrs later and subjected to analyze the dual luciferase activities with this system following the manufacturer’s protocol. The dual luciferase activities were measured by Varioskan Flash (Thermo Scientific). Luciferase activities were calculated by the ratio of the luminescence of firefly to renilla, and normalized to control.

***In vitro* tumor repopulation model**

The protocol of developing *in vitro* tumor repopulation model was described previously [2, 11]. Briefly, the cultured vigorous pancreatic cancer cells were exposed to 10Gy irradiation at 3.6Gy/min dose rate by using Oncor linear accelerator (Siemens, Germany). The irradiated cells were seeded into the 24-well culture plate at a density of 50,000 cells/well as feeder cells. Meanwhile, the luciferase-labeled living pancreatic cancer cells were immediately seeded into the co-culture system plate at a density of 1,000 cells/well as reporter cells. Aspirin was added to the culture media at a final concentration of 0.2mM. The fresh culture media containing 2% FBS was replaced every 3 days for 15 days. The substrate VivoGlo™ Luciferin (150 μg/ml, Promega) was added to the media before imaging. All the images were captured by IVIS Lumina Series III (Caliper LifeSciences) every 3 days for 15 days. The bioluminescent activities were analyzed by the image capture software.

**Cell cycle analysis**

Cell cycle analysis was performed by using BD Cycletest^TM^ Plus DNA Reagent Kit (BD Bioscience) according to the manufacturer’s protocol. In short, pancreatic cancer cells were transfected with miRNAs, lentivirus, or treated with exosomes, doxycycline or other reagents. Cells were collected by trypsinization and subjected to cell staining following the manufacturer’s protocol. The stained cells were further filtered with 70 μm nylon cell strainer (Falcon) and subjected to examine cell cycle by using BD LSRFortessa^TM^ Cell Analyzer. The ModFit LT 5.0 software was used to analyze the distribution of cell cycles.

**EdU cell proliferation assay**

The EdU cell proliferation assay was performed by using Cell-Light^TM^ EdU Apollo488 In Vitro Kit (RiboBio) according to the manufacturer’s instructions. In brief, EdU solution (1:1000) was added to the culture media of pancreatic cancer cells at the indicated time and cultured for 2 hrs. Then cells were fixed using 4% paraformaldehyde for 30 min and subjected to EdU staining. Images were captured by fluorescence microscope (Leica) and data were analyzed by using Image J software.

**Cell viability assay**

Cell viability assay was performed by using the CellTiter-Glo^®^ Luminescent Cell Viability Assay (Promega) following the manufacturer’s instructions. Briefly, pancreatic cancer cells were seeded into the 96-well opaque plate for routine culture. After 24 hrs, cells were exposed to radiation and/or treated with exosomes, aspirin or other reagents. Cells were further cultured for 24-72 hrs, and subjected to cell viability assay following the manufacturer's instructions. Images were taken by the IVIS Lumina Series III (Caliper LifeSciences). The bioluminescent activities were analyzed with the image capture software.

**Real-time cell analysis assay (RTCA)**

RTCA was performed by using xCELLigence RTCA DPlus (ACEA Bioscience) according to the manufacturer’s instructions. Briefly, cells were plated into E-Plate 16 (ACEA Bioscience) at 1,500 cells/well and kept at room temperature for 30 min for cell seeding. Cell proliferation was detected immediately after the plates were put into the machine. Data were collected every 15 min for the total time of 100 hrs. The cell proliferation data were then directly exported from the machine.

**Colony formation assay**

The ability of cell colony formation was detected by the plate colony forming assay. Briefly, to evaluate the plate colony formation ability of pancreatic cancer cells upon radiation, cells were seeded into 6-well plate at a density of 1×10^5^ cells/well or 12-well plate at 4×10^4^ in triplicate. For the unirradiated cells, we would just inoculate at 300 cells/well to 6-well plate or at 120 to 12-well plate. After 6 hrs incubation, cells were treated with GW4869 (10mM, SelleckChem), or transfected with miR-194-5p mimic (50nM, RiboBio), miR-194-5p inhibitor (100nM, RiboBio) or corresponding controls, and further exposed to 10Gy irradiation. To induce miR-194 expression under the control of tetracycline, the tetracycline derivative doxycycline (1μg/ml, MedChemExpress) was added to the media before 10Gy irradiation. Cell culture media were changed once every 3-4 days. After 12-14 days, the colonies were fixed using paraformaldehyde and stained by crystal violet. Colonies were scanned and counted by using Image J software.

The soft-agar colony formation experiment was used to evaluate the stemness of cancer cells. Briefly, the lower layer of the culture systems was formed by mixture of 1% agar and 2× culture media (2× DMEM/RPMI 1640 with 20% FBS) at the ratio of 1:1, while the upper layer was of 0.6% agar and 2× culture media with cells at a density of 5,000 cells/well in the 6-well plate. After agar solidification, 1ml of 1× culture media were added to each well to keep humidity. After 12-14 days, the colonies were stained by nitro-tetrazolium chloride blue (NBT) overnight. Colonies were scanned and counted by using Image J software.

**Wound healing assay**

The cell migration ability was evaluated with wound healing assay. In short, cells were plated into the 6-well plate at a density of 5×10^5^ cells/well and cultured for 24-48 hrs to reach around 100% confluence. The wounds were created using 200 μl tips by scratching. After scratching, the well was gently washed twice with medium to remove the detached cells. Images were taken at indicated times by EVOS XL Core (Life technologies).

**Transwell assay**

The transwell assay was applied to identify the invasion ability of cancer cells as previously described [2]. Briefly, cells were cultured in the hanging cell culture inserts with 8 μm pore size (PIEP12R48, Millipore) in 24-well plate. 20,000 cells suspended in 200 μl fresh media without FBS were added to the hanging cell culture inserts. 900 μl fresh media containing 10% FBS were added to the lower chamber. After 24 hrs culture, cells in the hanging cells were fixed with 4% paraformaldehyde, and stained with crystal violet. Cells in the inner side of the inserts were removed with cotton swabs. Images were taken by microscopy (Leica).

**Cancer stemness analysis**

Flow cytometry was applied to analyze the stemness of pancreatic cancer cells. Specifically, ALDH enzyme activities were detected by using the ALDEFLUOR™ kit (Stemcell Technology) following the manufacturer’s instructions. In brief, pancreatic cancer cells were cultured and treated as indicated. Then about 5×10^5^ cells were collected and washed twice with PBS and resuspended in 1 mL of ALDEFLUOR assay buffer. ALDEFLOUR reagent or ALDEFLOUR plus DEAB (ALDH enzyme inhibitor, as negative control) were added to the cell suspensions, mixed and incubated in 37 ℃ water baths for 40 min. 20,000 viable cells each group were analyzed by the BD LSRFortessa^TM^ Cell Analyzer. Data were analyzed using FlowJo V10 software.

CD24 and CD326 (ESA) were also detected by flow cytometry. Briefly, 1×10^6^ cells were collected and washed twice with PBS and FACS buffer (1× PBS, 2% FBS, 0.1% NaN_3_), and then resuspended in 100 μl FACS buffer. PE Mouse anti-Human CD24 (BD Bioscience, 560991), BB515 Mouse anti-Human CD326 (BD Bioscience, 565398) were added to the cell suspensions at the recommended concentration and incubated for 10 min at room temperature. Cells were further subjected to flow cytometry analysis by the BD LSRFortessa^TM^ Cell Analyzer. Data were analyzed using FlowJo V10 software.

**Comet assay**

DNA strand breaks were evaluated by alkaline single cell gel electrophoresis (comet assay) according to the protocol described before [12]. Briefly, 400 μl single cell suspension in PBS was mixed with 1200 μl of 1% low gelling temperature agarose (sigma), transferred onto slides precoated with 1% gel. Cells in the gel were incubated for 6 hrs at 4 °C in the alkaline lysis solution (1.2 M NaCl, 100 mM EDTA, 0.1% sodium lauryl sarcosinate, 0.26 M NaOH) and then electrophoresed in the electrophoresis solution (0.03 M NaOH, 2 mM EDTA) for 25 min at 14 V voltage. Cells were then stained with 0.25 μg/ml propidium iodide (sigma). % tail DNA (tail DNA/Total DNA) of each cell was calculated using Leica LAS X.

**H&E staining and immunohistochemistry**

H&E staining and immunohistochemistry were performed for morphological evaluation as described [13]. In short, fresh resected tissues were fixed with 4% paraformaldehyde at room temperature for 12-24 hrs, embedded in paraffin. Paraffin tissue blocks were cut into 4 μm thick sections using a standard sliding manual microtome (Leica). The sections were mounted onto glass slides. H&E staining was done with Leica AutoStainer XL according to the operation manual. Images were taken by microscopy (Leica).

All sections were subjected to antigen retrieval before immunohistochemical staining. Antigen retrieval was achieved with heat in boiling citrate antigen retrieval solution (Sangon Biotech) for 10 min under microwave. Sections was blocked using 3% H_2_O_2_ for 15 min, and immunostaining blocking/primary antibody dilution buffer (Sangon Biotech) for 1 hr. The blocked sections were then incubated overnight at 4 ℃ with the primary antibodies, anti-ALDH1A1 (1:200, CST 12282) , anti-Phospho-Histone H2A.X (Ser139) (1:200, CST 80312) , anti-Ki-67 (1:100, Abcam ab16667), anti-GFP (1:100, Abcam ab5450). Immunohistochemistry was done by using the GTvision Immunohistochemistry Detection Kit (Gene Tech, 5007) according to the manufacturer’s protocol. For immunofluorescence staining, the secondary antibodies, Alexa Fluor 488 AffiniPure Donkey anti-Goat IgG (H+L) (Yeasen, 34306ES60), Anti-mouse IgG (H+L), F(ab')2 Fragment (Alexa Fluor® 594 Conjugate) (CST, 8890), Anti-mouse IgG (H+L), F(ab')2 Fragment (Alexa Fluor® 488 Conjugate) (CST, 4408), Anti-rabbit IgG (H+L), F(ab')2 Fragment (Alexa Fluor® 488 Conjugate) (CST, 4412), Anti-rabbit IgG (H+L), F(ab')2 Fragment (Alexa Fluor^®^ 594 Conjugate) (CST, 8889), were used for visualization under the fluorescence microscope (Leica) or the laser confocal scanning microscope (Leica).

The cultured cells upon different treatments were also subjected to immunofluorescence analysis. Briefly, cells were fixed with 4% paraformaldehyde at room temperature for 30 min and permeabilized with 0.1% Triton X-100. Cells were blocked using blocking buffer for 1 hrs and then incubated overnight at 4 ℃ with the primary antibodies, anti-GFP (1:100, Abcam ab5450) and anti-Phospho-Histone H2A.X (Ser139) (1:200, CST 80312). The subsequent procedures were the same as the immunofluorescence staining of tissue sections.

**Exosomal miRNA sequencing data analysis**

Raw data were generated after sequencing, image analysis, base calling and quality filtering on Illumina sequencer. Firstly, Q30 was used to perform quality control. The adaptor sequences were trimmed and the adaptor-trimmed-reads (≥15nt) were left by cutadapt software (v1.9.3). Then, trimmed reads from all samples were pooled, and miRDeep2 software (v2.0.0.5) was used to predict novel miRNAs. The trimmed reads were aligned to the merged human pre-miRNA databases (known pre-miRNA from miRBase plus the newly predicted pre-miRNAs) using Novoalign software (v3.02.12) with at most one mismatch. The numbers of mature miRNA mapped tags were defined as the raw expression levels of that miRNA. The read counts were normalized by TPM (tag counts per million aligned miRNAs) approach. Differentially expressed miRNAs between two samples were filtered through fold change. Differentially expressed miRNAs between two groups were filtered by fold change and p-value.

**Cellular RNA sequencing data analysis**

FastQC (version 0.11.2) was used for evaluating the quality of sequenced data. Raw reads were filtered by Trimmomatic (version 0.36). Clean reads were mapped to the reference genome by HISAT2 (version 2.0) with default parameters. RSeQC (version 2.6.1) was used to statistics the alignment results. The homogeneity distribution and the genome structure were checked by Qualimap (version 2.2.1). BEDTools (version 2.26.0) was used to statistically analyze the gene coverage ratio. Gene expression values of the transcripts were computed by StringTie (version 1.3.3b). DESeq2 (version 1.12.4) was used to determine differentially expressed genes (DEGs) between two samples. Genes were considered as significant differentially expressed if q-value <0.001 and fold change >2. When the normalized expression of a gene was zero between two samples, its expression value was adjusted to 0.01 (as 0 cannot be plotted on a log plot). If the normalized expression of a certain gene in two libraries was all lower than 1, further differential expression analysis was conducted without this gene. Functional enrichment analyses including Gene Ontology (GO) was performed to identify which DEGs were significantly enriched in GO terms or metabolic pathways. GO terms with false discovery rate (q-value) <0.05 were considered as significantly altered.

**Statistics**

All data were analyzed with software GraphPad Prism 7. Normally distributed data were presented as mean with SD. Differences between means were assessed using unpaired student’s t test. p<0.05 was considered statistically significant. Pearson correlation coefficient (r value) was calculated assuming linear relationship between variables.

**References**

1. Gu DN, Jiang MJ, Mei Z, Dai JJ, Dai CY, Fang C, Huang Q, Tian L: microRNA-7 impairs autophagy-derived pools of glucose to suppress pancreatic cancer progression**.** Cancer Lett 2017, 400**:**69-78.

2. Fang C, Dai CY, Mei Z, Jiang MJ, Gu DN, Huang Q, Tian L: microRNA-193a stimulates pancreatic cancer cell repopulation and metastasis through modulating TGF-beta2/TGF-betaRIII signalings**.** J Exp Clin Cancer Res 2018, 37**:**25.

3. Dinkins MB, Dasgupta S, Wang G, Zhu G, Bieberich E: Exosome reduction in vivo is associated with lower amyloid plaque load in the 5XFAD mouse model of Alzheimer's disease**.** Neurobiol Aging 2014, 35**:**1792-1800.

4. Zelenay S, van der Veen AG, Bottcher JP, Snelgrove KJ, Rogers N, Acton SE, Chakravarty P, Girotti MR, Marais R, Quezada SA, et al: Cyclooxygenase-Dependent Tumor Growth through Evasion of Immunity**.** Cell 2015, 162**:**1257-1270.

5. Matsuda T, Cepko CL: Controlled expression of transgenes introduced by in vivo electroporation**.** Proc Natl Acad Sci U S A 2007, 104**:**1027-1032.

6. D'Astolfo DS, Pagliero RJ, Pras A, Karthaus WR, Clevers H, Prasad V, Lebbink RJ, Rehmann H, Geijsen N: Efficient intracellular delivery of native proteins**.** Cell 2015, 161**:**674-690.

7. Doench JG, Fusi N, Sullender M, Hegde M, Vaimberg EW, Donovan KF, Smith I, Tothova Z, Wilen C, Orchard R, et al: Optimized sgRNA design to maximize activity and minimize off-target effects of CRISPR-Cas9**.** Nat Biotechnol 2016, 34**:**184-191.

8. Sanson KR, Hanna RE, Hegde M, Donovan KF, Strand C, Sullender ME, Vaimberg EW, Goodale A, Root DE, Piccioni F, Doench JG: Optimized libraries for CRISPR-Cas9 genetic screens with multiple modalities**.** Nat Commun 2018, 9**:**5416.

9. Sanjana NE, Shalem O, Zhang F: Improved vectors and genome-wide libraries for CRISPR screening**.** Nat Methods 2014, 11**:**783-784.

10. Thery C, Amigorena S, Raposo G, Clayton A: Isolation and characterization of exosomes from cell culture supernatants and biological fluids**.** Curr Protoc Cell Biol 2006, Chapter 3**:**Unit 3 22.

11. Huang Q, Li F, Liu X, Li W, Shi W, Liu FF, O'Sullivan B, He Z, Peng Y, Tan AC, et al: Caspase 3-mediated stimulation of tumor cell repopulation during cancer radiotherapy**.** Nat Med 2011, 17**:**860-866.

12. Olive PL, Banath JP: The comet assay: a method to measure DNA damage in individual cells**.** Nat Protoc 2006, 1**:**23-29.

13. Fischer AH, Jacobson KA, Rose J, Zeller R: Hematoxylin and eosin staining of tissue and cell sections**.** CSH Protoc 2008, 2008**:**pdb prot4986.
