# Supplementary material for: Dying tumor cell-derived exosomal miR-194-5p potentiates survival and repopulation of tumor repopulating cells upon radiotherapy in pancreatic cancer
Source: Mol Cancer. 2020 Mar 30;19:68. doi: 10.1186/s12943-020-01178-6 (PMC7104536; doi:10.1186/s12943-020-01178-6)
Supplement: Supplementary file 11 — Additional file 11:Table S1. Oligos and primers used in this study. [file 12943_2020_1178_MOESM11_ESM.docx]

**Supplementary Table S1.** Oligos and primers used in this study.

| **Oligonucleotides** | **Supplier** | **Catalog number or sequence** |
| --- | --- | --- |

| micrON hsa-miR-194-5p mimic | RiboBio | Cat# miR10000460-1 |
| --- | --- | --- |

| micrON mimic NC #22 | RiboBio | Cat# miR1N0000001-1 |
| --- | --- | --- |
| micrOFF hsa-miR-194-5p inhibitor | RiboBio | Cat# miR20000460-1 |
| micrOFF inhibitor NC #22 | RiboBio | Cat# miR2N0000001-1 |
| ALDH1A1 forward primer | Sangon Biotech | 5’-GTACTAGTCCTGCTGGCTTTTCTGTTCAC-3’ |
| ALDH1A1 reverse primer | Sangon Biotech | 5’-GTGAATTCTTCTGATTCGGCTCCTGGAAC-3’ |
| HMGA2 forward primer | Sangon Biotech | 5’-TAGGATCCGGGAGGCAGGATGAGCGCAC-3’ |
| HMGA2 reverse primer | Sangon Biotech | 5’-CAGCTAGCAGAAATCGAACGTTGGCGCC-3’ |
| E2F3 forward primer | Sangon Biotech | 5’-GACGGATCCGAGCAGGAGCGAGAGATGAG-3’ |
| E2F3 reverse primer | Sangon Biotech | 5’-CTGCTAGCGAGTTCACACGAAGCATAATC-3’ |
| MIR194 forward primer | Sangon Biotech | 5’-ACGGATCCGACCAATGACAGTAAAGTCAG-3’ |
| MIR194 reverse primer | Sangon Biotech | 5’-CTGGCTAGCTCCTCTTCCATTATTGGATCT-3’ |
| sgHMGA2-1 forward oligo | Sangon Biotech | 5’-CACCGGCCCTCTCCTAAGAGACCCA-3’ |
| sgHMGA2-1 reverse oligo | Sangon Biotech | 5’-AAACTGGGTCTCTTAGGAGAGGGCC-3’ |
| sgHMGA2 forward oligo | Sangon Biotech | 5’-CACCGGGAGGCAGGATGAGCGCACG-3’ |
| sgHMGA2-2 reverse oligo | Sangon Biotech | 5’-AAACCGTGCGCTCATCCTGCCTCCC-3’ |
| sgE2F3-1 forward oligo | Sangon Biotech | 5’-CACCGGACCTCAAACTGTTAACCG-3’ |
| sgE2F3-1 reverse oligo | Sangon Biotech | 5’-AAACCGGTTAACAGTTTGAGGTCC-3’ |
| sgE2F3-2 forward oligo | Sangon Biotech | 5’-CACCGAGTCTAAAAACAACGTCCAA-3’ |

| sgE2F3-2 reverse oligo | Sangon Biotech | 5’-AAACTTGGACGTTGTTTTTAGACTC-3’ |
| --- | --- | --- |

| E2F3 3’UTR forward oligo | Sangon Biotech | 5’-CATGCTAGCGATTATGCTTCGTGTGAACTC-3’ |
| --- | --- | --- |
| E2F3 3’UTR reverse oligo | Sangon Biotech | 5’-CTAGTCGACCATTTTATTGATCCTTTACCA-3’ |
| 194PC forward oligo | Sangon Biotech | 5’-CTAGTCCACATGGAGTTGCTGTTACA-3’ |
| 194PC reverse oligo | Sangon Biotech | 5’-TCGATGTAACAGCAACTCCATGTGGA-3’ |
| E2F3 WT forward oligo | Sangon Biotech | 5’-CTAGTGGATTTTGTAAATAGATTTGTTACAGGGTGA-3’ |
| E2F3 WT reverse oligo | Sangon Biotech | 5’-TCGATCACCCTGTAACAAATCTATTTACAAAATCCA-3’ |
| E2F3 MT forward oligo | Sangon Biotech | 5’-CTAGTGGATTTTGTAAATAGATTGTACTTAGGGTGA-3’ |
| E2F3 MT reverse oligo | Sangon Biotech | 5’-TCGATCACCCTAAGTACAATCTATTTACAAAATCCA-3’ |
| miR-194 reverse transcription primer | Sangon Biotech | 5’-GTCGTATCCAGTGCAGGGTCCGAGGTATTCGCACTGGATAC GACTCCACA-3’ |
| RUN6B reverse transcription primer | Sangon Biotech | 5’-CTCAACTGGTGTCGTGGAGTCGGCAATTCAGTTGAGAAAA ATAT-3’ |
| miR194 forward primer for qPCR | Sangon Biotech | 5’-CGCGTGTAACAGCAACTCCA-3’ |
| miR194 reverse primer for qPCR | Sangon Biotech | 5’-AGTGCAGGGTCCGAGGTATT-3’ |
| RUN6B forward primer for qPCR | Sangon Biotech | 5’-CAAGGATGACACGCAAA-3’ |
| RUN6B reverse primer for qPCR | Sangon Biotech | 5’-TCAACTGGTGTCGTGG-3’ |
| HMGA2 forward primer for qPCR | Sangon Biotech | 5’-AAGCAGAAGCCACTGGAGAA-3’ |
| HMGA2 reverse primer for qPCR | Sangon Biotech | 5’-CGGCAGACTCTTGTGAGGAT-3’ |
| E2F3 forward primer for qPCR | Sangon Biotech | 5’-GTCATCAGTACCTCTCAGATGG-3’ |
| E2F3 reverse primer for qPCR | Sangon Biotech | 5’-GCAGACCAAGAGACGTATCATA-3’ |

| ALDH1A1 forward primer for qPCR | Sangon Biotech | 5’-ACGCCAGACTTACCTGTCCTACTC-3’ |
| --- | --- | --- |

| ALDH1A1 reverse primer for qPCR | Sangon Biotech | 5’-GCCTTGTCAACATCCTCCTTATCTCC-3’ |
| --- | --- | --- |
| CD133 forward primer for qPCR | Sangon Biotech | 5’-CACTACCAAGGACAAGGCGTTCAC-3’ |
| CD133 reverse primer for qPCR | Sangon Biotech | 5’-TCTTCAAGGTGCTGTTCATGTTCTCC-3’ |
| CD44 forward primer for qPCR | Sangon Biotech | 5’-ACAACGCAGCAGAGTAATTCTCAGAG-3’ |
| CD44 reverse primer for qPCR | Sangon Biotech | 5’-TCCACCTGTGACATCATTCCTATTGC-3’ |
| TSG101 forward primer for qPCR | Sangon Biotech | 5’-TCCATATCCTGCCACAACAAGTTCTC-3’ |
| TSG101 reverse primer for qPCR | Sangon Biotech | 5’-TCCTCCTTCATCCGCCATCTCAG-3’ |
| CD63 forward primer for qPCR | Sangon Biotech | 5’-ACAGAGTTGGAGCCAGAGGAGAG-3’ |
| CD63 reverse primer for qPCR | Sangon Biotech | 5’-GTTCAGAGAAGCGGACGAGGTG-3’ |
| CD81 forward primer for qPCR | Sangon Biotech | 5’-TCGCCAAGGATGTGAAGCAGTTC-3’ |
| CD81 reverse primer for qPCR | Sangon Biotech | 5’-GCCACAGCAGTCAAGCGTCTC-3’ |
| GAPDH forward primer for qPCR | Sangon Biotech | 5’-GGGAAGGTGAAGGTCGGAGT-3’ |
| GAPDH reverse primer for qPCR | Sangon Biotech | 5’-GGGGTCATTGATGGCAACA-3’ |
